# On-chip nonlocal metasurface for color router: conquering efficiency-loss from spatial-multiplexing

**DOI:** 10.1038/s41377-025-02146-9

**Published:** 2026-01-12

**Authors:** Yangyang Shi, Shuai Wan, Zejing Wang, Runlong Rao, Zhongyang Li

**Affiliations:** 1 Electronic Information School, Wuhan University, Wuhan, 430072 China; 2 Wuhan Institute of Quantum Technology, Wuhan, 430206 China

**Keywords:** Metamaterials, Sub-wavelength optics, Nanophotonics and plasmonics

## Abstract

Metasurfaces integrated onto guided-wave photonic systems have been investigated for enabling advanced functionalities such as point-by-point optical extraction and manipulation of amplitude, phase, and polarization. However, achieving full control over the spectrum (i.e., wavelength/frequency) of on-chip light remains a challenge, limiting their widespread application in integrated photonics. Here, we propose and experimentally demonstrate an on-chip metasurface color router by leveraging symmetry-broken quasi-bound states in the continuum (q-BICs) mode. By precisely engineering the on-chip meta-diatom pairs with controlled scaling and asymmetry, we simultaneously achieve modulation of both extraction intensity and narrowband spectral extraction of the out-coupled lightwave. As a proof of concept, we realize several on-chip multiplexed color routers through spatial mapping and cascading of distinct q-BIC-assisted meta-diatom pixels, capable of selectively guiding and routing primary wavelengths into free space from different spatial positions along the waveguide. Crucially, due to the on-chip optical propagation scheme, these color routers, enabled by nonlocal metasurfaces, exhibit spatial multiplexing but with a significant improvement in the energy utilization efficiency (EUE) compared with conventional designs. We envision that such on-chip q-BIC-assisted metasurface color routers, with their potential for miniaturized integration, could open new avenues for advanced applications in multiplexed information routing, intelligent integrated photonic systems, and next-generation wearable display technologies.

## Introduction

Toward developing chip-integrated and miniaturized nanophotonic devices, the incorporation of metasurfaces onto optical waveguides has attracted widespread attention as a compact platform for manipulating and guiding the in-plane lightwave^1^. Recently, through meticulously patterning subwavelength nanostructures onto the waveguide, on-chip integrated metasurfaces have enabled precise control of the amplitude, phase, and polarization of the extracted light and achieved versatile functionalities^2–4^, including directional beam-steering^5–7^, on-chip metalens^8–10^, vortex beam generator^11–13^, meta-holography^14–19^, and screen display^20^, etc. Due to the on-chip propagation scheme, this technology enjoys the unique merits of no zeroth-order background interference, capability of multiple cascading, and compatibility with other miniature on-chip elements, which could facilitate the creation of advanced chip-integrated meta-optics devices.

On-chip metasurfaces have demonstrated considerable success in phase modulation, leveraging mechanisms such as resonant phase^5,10,14^ or detour phase mechanisms^16–18^; amplitude modulation has been achieved through meta-atom size^21^ or meta-diatom interference^20,22^; polarization control has utilized geometric phase^23–25^ along with other phase mechanisms^15,20,26–30^. Despite the above advances, these approaches offer limited control over the spectral properties of the out-coupling light, with insufficient capability to achieve wavelength-selective light extraction. Recent advancements in on-chip metasurfaces, along with their comparative analysis, are detailed in Supplementary Table S1, Supplementary Information. Typically, when guided waves are perturbed by on-chip meta-atoms, the extracted light exhibits a broadband response with minimal wavelength selectivity^16^. More details can be found in Supplementary Fig. S1, Supplementary Information. To date, there remains no viable strategy for precise wavelength-selective extraction for on-chip meta-systems.

The ability to modulate wavelength while controlling the extraction selectively on an on-chip platform is crucial for advancing integrated photonic applications, such as wavelength-division multiplexing (WDM) and color routing. More discussion can be found in Supplementary Section S1, Supplementary Information. Thus, unlocking the additional degrees of freedom necessary for wavelength-selective on-chip extraction represents a key challenge and open frontier in this field. It is worth mentioning that conventional free-space metasurfaces or multilayer thin films readily enable color filters and routing functionality^31–34^. However, these approaches suffer from significant energy utilization efficiency (EUE) losses due to spatial multiplexing, as a considerable portion of the incident light is wasted outside the passband of each wavelength filter. Consequently, an efficient solution tailored for an on-chip platform to achieve wavelength-selective extraction but without the significant EUE losses associated with spatial multiplexing, has yet to be realized.

In this work, we propose and experimentally demonstrate an on-chip nonlocal metasurface that supports symmetry-protected bound states in the continuum (BICs) integrated with a waveguide for wavelength-selective extraction and color routing functionality. By precisely tuning the geometric asymmetry and scaling factor of on-chip meta-diatom pairs, we achieve selective extraction and routing of guided waves into free space, resulting in sharp spectral extraction with tunable intensity. As a proof of concept, we implement spatial mapping and horizontally cascade distinct quasi-BIC (q-BIC)-assisted pixels to demonstrate multicolor routing, encoding both color and intensity information into meta-diatom pairs with varying geometric dimensions. Compared to conventional free-space metasurface routers, these on-chip color routers utilize cascading multiplexing but without incurring significant losses in EUE. Additionally, the generated multicolor meta-displays effectively suppress zeroth-order background interference, owing to the on-chip propagation scheme. Overall, we envision that the on-chip nonlocal metasurface platform, with its advantages in miniaturized integration, holds significant potential for advanced applications in next-generation wearable meta-display devices, WDM, and information routing in integrated photonic systems.

## Results

**Fig. 1:**
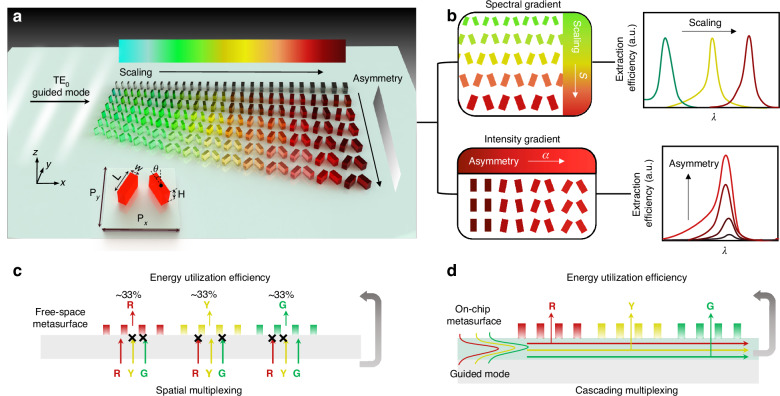
Schematic illustration of on-chip color routing metasurfaces based on q-BICs-inspired physics. **a** The on-chip q-BIC-assisted metasurface is integrated onto the waveguide to extract and route the guided waves, illustrated by a color spectral gradient along the *x*-direction and an intensity gradient along the *y*-direction. **b** The spectral selection and intensity of the extracted guided waves can be controlled by engineering the scaling factor and tilting angle of the meta-diatoms, respectively. Inset: Perspective view of the unit cell of the on-chip nonlocal metasurface composed of two tilting *α*-Si nanoblocks. **c**, **d** Schematic illustrating the distinction between on-chip cascading multiplexing and conventional free-space spatial multiplexing color routers

Figure 1a schematically illustrates the on-chip q-BIC-assisted metasurface color router with intensity tunability patterned onto the waveguide configuration. Here, the on-chip metasurface is endowed with dual gradient characteristics, that is, a scaling factor of *S* to modify the dimensions of each unit cell (*x*-direction) and an asymmetry factor of *α* to vary the tilting angle *θ* of each pair of nanoblocks (*y*-direction). The fundamental TE_0_ mode guided waves propagating along the *x*-direction are extracted by the on-chip q-BIC-assisted metasurface into free space to generate and route vibrant and brightness-tunable colors. Specifically, by continuously varying the scaling factor *S*, the spectral tuning of the extracted guided waves is achieved, thus creating spectral gradients (Fig. 1b). Meanwhile, by arranging unit cells with the identical *S* but varying *θ* into a 2D array, we could generate an extraction intensity gradient at a specific extracted wavelength. In contrast to previous on-chip metasurfaces with broadband and non-selective wavelength extraction, the proposed on-chip nonlocal metasurface can extract a narrowband (~20 nm on average) of guided light with a tailored wavelength peak and intensity into free space, extending the degrees of freedom to manipulate the frequency and amplitude of the guided waves.

In contrast, free-space metasurface color filters or routers based on spatial multiplexing allow narrowband light to pass through and other wavelength components to be discarded, thus suffering from the intrinsic EUE upper limit, as shown in Fig. 1c. Here, EUE (defined as the ratio of the transmitted light intensity of a single-wavelength channel to that of the total incident light) is constrained to an upper limit of ~33% (for three color channels). However, our q-BIC-assisted diatom pairs benefiting from an on-chip cascade propagation scheme, extract the respective narrowband guided light without energy waste from other wavelengths, as illustrated in Fig. 1d. This design overcomes the theoretical limitation of conventional spatial multiplexing EUE, potentially achieving an EUE value close to unity, assuming minimal propagation losses and optical absorption losses. More details on EUE comparisons and simulation results are provided in Supplementary Section S2, Supplementary Information.

The proposed on-chip nonlocal metasurface consists of pairs of tilted amorphous silicon (*α*-Si) nanoblocks with a long-axis *L* and a short-axis *W* (as shown in the inset of Fig. 1a), which are on top of a planar waveguide of Si_3_N_4_ (thickness = 220 nm) with a high effective index (*n* = ~2.05) with a thick (~500 μm) SiO_2_ substrate underneath. More details on the refractive index of the silicon material can be found in Supplementary Section S3, Supplementary Information. Here, the height *H* of the meta-diatoms is 380 nm, and *θ* is the tilting angle of the long axis of the meta-atom with respect to the *y*-axis. The periods along the *x*- and *y*-directions are denoted as *P*_*x*_ and *P*_*y*_, respectively.

**Fig. 2:**
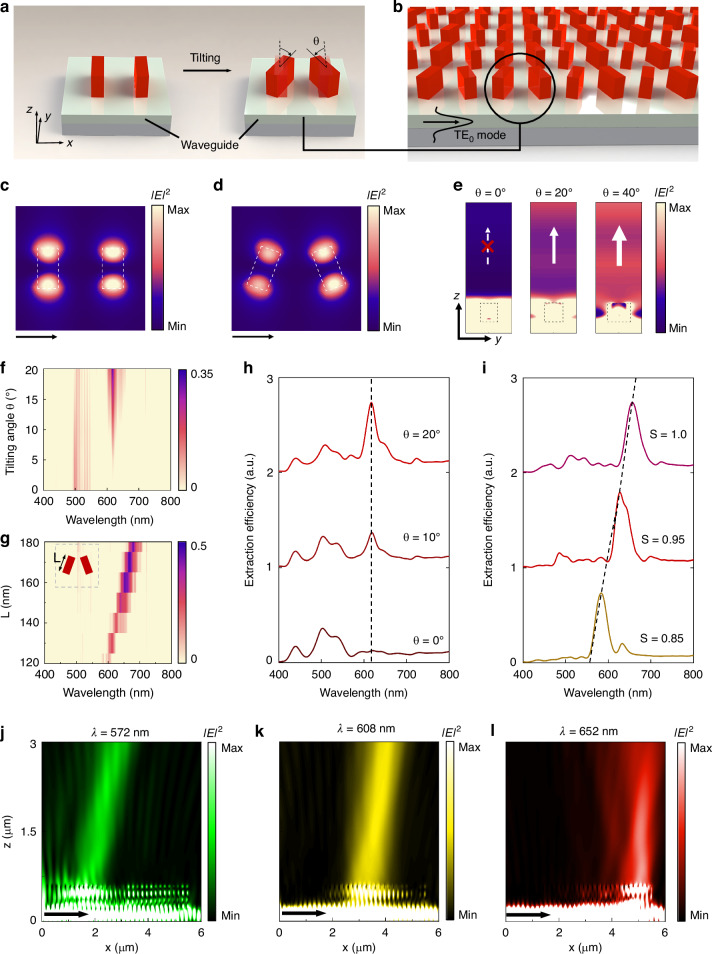
Design principle and numerical analysis of the on-chip q-BIC-assisted metasurface for color routing and intensity tuning. **a** 3D schematic of on-chip diatomic pixels consisting of two parallel and two tilted nanoblocks, respectively, corresponding to the conversion from a BIC mode to a q-BIC mode. Here, *θ* is the tilting angle of the q-BIC meta-diatom. The local displacement between diatomic nanoblocks along the *x*-direction is designed to be *P*_*x*_/2. **b** Schematic of on-chip q-BIC-assisted metasurface composed of pairs of tilted rectangular nanoblocks integrated onto the waveguide. **c**, **d** Simulated electric-field intensity (|*E* | ^2^) distributions in the *xy*-plane for the two cases of tilting angle *θ* = 0° and *θ* = 20° when the TE_0_ mode incidents from the *x*-direction. The corresponding parameters are *L* = 130 nm, *W* = 65 nm, *P*_*x*_ = *P*_*y*_ = 400 nm, and *H* = 380 nm. **e** Electric-field intensity distributions (|*E*|^2^) in the *yz*-plane for the case of tilting angle *θ* = 0°, *θ* = 20°, and *θ* = 40°. **f** Simulated extraction spectrum contour of the on-chip BIC or q-BIC meta-diatomic array as the tilting angle varies from 0° to 20°. **g** Simulated extraction spectra of q-BIC meta-diatomic array with length *L* varying from 120 nm to 180 nm. The corresponding parameters are *W* = 60 nm, *P*_*x*_ = *P*_*y*_ = 400 nm, and *H* = 380 nm. **h** Line plot of extraction spectra of BIC or q-BIC meta-diatomic array for the case of tilting angle *θ* = 0°, *θ* = 10°, and *θ* = 20°, respectively, showing that the Q-factors vary from 23 to 18 as the angle *θ* increases from 10° to 20°. **i** Line plot of extraction spectra of q-BIC meta-diatomic array with the scaling factors *S* (as given by *L* = *L*_0_ × *S*, *W* = *W*_0_ × *S*, *P* = *P*_0_ × *S*) varying from 0.85 to 1 while keeping *θ* fixed at 20°. The average Q-factor is ~17. The corresponding parameters are *L*_0_ = 160 nm, *W*_0_ = 60 nm, and *P*_0_ = 400 nm when *S* = 1. **j**–**l** Simulated electric-field intensity (|*E*|^2^) profiles extracted from the waveguide at the wavelength of 572 nm, 608 nm, and 652 nm. The black arrows represent the propagation direction of the guided waves

The key operating principle of our on-chip color routing metasurfaces is based on the concept of q-BICs^35–40^. Through leveraging destructive mode coupling or breaking symmetry in parameter and momentum spaces to induce the leaky q-BIC mode, nonlocal metasurfaces can achieve finite high quality factors (Q-factors) and have shown promise in applications such as spatial radiation tailoring^41^, Q-factor enhancement^42^, and group delay optimization^43^. Here, by applying spatially varying perturbations to break the in-plane inversion symmetry of meta-diatom (Fig. 2a), it can convert guided optical modes (bound waves) into customized free-space optical fields (quasi-bound waves), thus shaping the extracted spectra. Specifically, the symmetry-protected BIC structure (*θ* = 0°) is in an unperturbed state with a large wavevector, which confines the guided wave to propagate inside the waveguide instead of being extracted into free space. As asymmetry is gradually induced (*θ* > 0°), the perturbation is applied to the meta-diatomic pair, doubling the effective lattice period, thus rendering the guided mode leaked into free space due to a period-doubling perturbation. More details on the operating principle of our on-chip nonlocal metasurfaces can be found in Supplementary Section S4, Supplementary Information.

Figure 2b presents the 3D schematic of the designed q-BIC antenna array at TE_0_ mode incidence condition. The simulated in-plane electric field intensity |*E*|^2^ profiles of meta-diatoms with *θ* = 0° (Fig. 2c) and *θ* = 20° (Fig. 2d) present the mode patterns of BIC and q-BIC being excited on the periodic array, respectively. The photonic leaky behavior of symmetry-protected metasurfaces is fundamentally determined by the asymmetry factor *α*, defined as the sine of the tilting angle *θ*, that is, *α* = sin(*θ*). The asymmetry factor *α* controls the extracted light intensity, allowing spectral tuning in intensity via varying the tilting angle *θ*. The simulated electric-field intensity (|*E*|^2^) distributions at the *yz*-cross sections of meta-diatoms with *θ* = 0°, 20°, and 40° (Fig. 2e) showcase notable intensity variation, indicating that increased asymmetry produces stronger off-chip extraction. Originating in BIC-inspired physics, the simulated 2D extraction spectra shown in Fig. 2f, as a function of *θ*, exhibit a sharp peak around 618 nm. The detailed extraction spectra in Fig. 2h show a corresponding increase in extraction intensity as the tilting angle *θ* increases from 0° to 20°, confirming that tuning the extraction spectrum intensity can be achieved by adjusting the asymmetry parameter.

In addition to the intensity control, wavelength-selective extraction of the out-coupling light can also be simultaneously achieved by tailoring the meta-diatom dimension. As the meta-diatomic length *L* increases from 120 nm to 180 nm (with a fixed width of 60 nm), the simulated extraction spectral peak position redshifts from 602 nm to 680 nm (Fig. 2g). We further numerically investigate the dependence of the extraction spectra on the unit cell dimensions multiplied by a scaling factor *S* (as given by *L* = *L*_0_ × *S*, *W* = *W*_0_ × *S*, *P* = *P*_0_ × *S*), which reveals that adjusting *S* from 0.85 to 1 for *θ* = 20° shifts the spectral peak from 580 nm to 660 nm (Fig. 2i). More details on the numerical simulations of far-field intensity profiles can be found in Supplementary Section S5, Supplementary Information.

To more intuitively illustrate the unique color routing capabilities, we predefine a continuous gradient of meta-diatomic length *L* varying from 95 nm to 170 nm (keeping *W* fixed at 60 nm and the tilting angle *θ* fixed at 20°) to arrange the q-BIC-assisted meta-diatom pairs along the propagation direction (*x*-axis). The simulated electric-field intensity |*E*|^2^ profiles at *xz*-cross sections of gradient meta-diatom array (Fig. 2j–l), exhibit that guided waves with different wavelength components would be strongly extracted from respective different locations into free space. Therefore, such on-chip cascading could selectively extract guided waves of different wavelengths from different spatial positions into free space for color routing. More details on the numerical simulations of on-chip color routing can be found in Supplementary Section S6, Supplementary Information, as well as on-chip q-BIC-assisted arrays with a gradient variation in the tilting angle presented in Supplementary Section S7, Supplementary Information. In addition, we can achieve wavelength-selective extraction and color routing functionalities covering the blue light band in the simulation, and more details can be found in Supplementary Section S8, Supplementary Information. We also further investigated the extraction performance of additional alternative on-chip meta-atom arrays with different materials, geometrical configurations, and resonance mechanisms. More details are provided in Supplementary Sections S9 and S10, Supplementary Information.

**Fig. 3:**
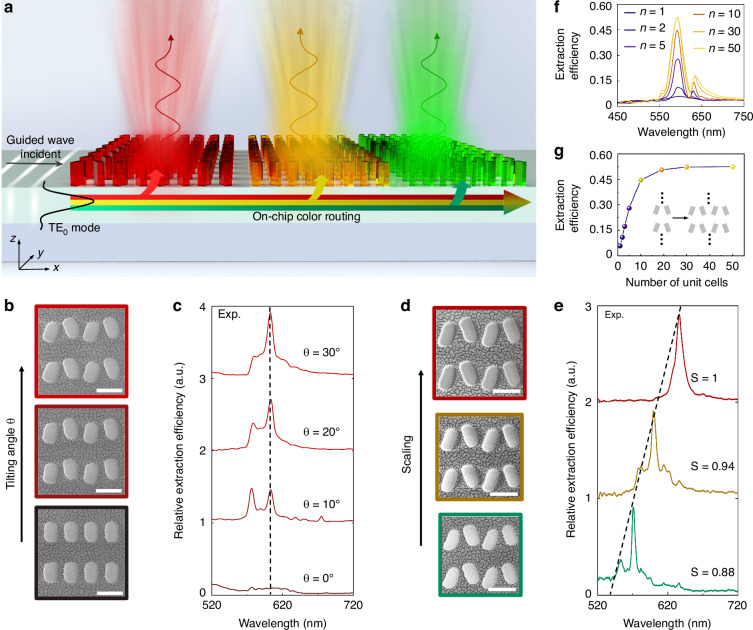
Characterization of on-chip q-BIC-assisted metasurface for color routing and intensity tuning. **a** Schematic of the concept of on-chip q-BIC-assisted metasurface composed of distinct arranged meta-diatomic pairs for color routing. **b** SEM image of the fabricated on-chip metasurface sample with varying tilting angle *θ* from 0° to 20°. Scale bar: 250 nm. **c** The experimental spectra of extracted light from different meta-diatomic pairs with the tilting angle *θ* varying from 0° to 30°, showing that the Q-factors vary from 64 to 46 as the angle *θ* increases from 10° to 30°. The corresponding parameters are *L*_0_ = 90 nm, *W*_0_ = 50 nm, and *P*_0_ = 374 nm. **d** SEM image of the fabricated on-chip metasurface sample with the scaling factor *S* increases. Scale bar: 250 nm. **e** The experimental extraction spectra from different meta-diatom pairs with the scaling factor *S* varying from 0.88 to 1. The corresponding Q-factors are 95 (green), 67 (yellow), and 53 (red), respectively. The corresponding parameters are *L*_0_ = 100 nm, *W*_0_ = 60 nm, and *P*_0_ = 400 nm while keeping *θ* fixed at 25°. **f** Simulated extraction spectra of the different sized arrays with number *N* along the propagation direction (*x*-axis) varying from 1 to 50. **g** Plot of maximum extracted efficiency from the spectra in (**f**)

Beyond free-space metasurfaces, on-chip metasurfaces offer distinct advantages to horizontally cascade multiple structures. This allows for the integration of periodic q-BIC-assisted arrays to selectively extract and route guided waves, as conceptually shown in Fig. 3a. As mentioned in Fig. 1d, when broadband guided light encounters a red-color extraction array, light waves of other colors (e.g., yellow and green) continue propagating without EUE loss. It is noteworthy that the on-chip extraction efficiency is relatively low (less than ~7%) per se^16,20,26^; however, the emphasis of no EUE loss here refers to that, thanks to the on-chip cascading strategy, all extracted energy is fully utilized to construct the corresponding wavelength channel, without any energy block or waste due to the conventional spatial-multiplexing scheme, assuming minimal propagation losses and optical absorption losses. In addition, considering realistic losses, the calculated EUE value from our simulation is ~0.62, which is nearly twice the theoretical upper limit of ~33% for three color channels in free-space alternatives. Overall, the extraction efficiency and EUE are fundamentally different concepts, and the high EUE achieved in our work should not be interpreted as an enhancement of the absolute extraction efficiency over previously reported on-chip metasurfaces. More details can be found in Supplementary Sections S2 and S11, Supplementary Information.

To experimentally demonstrate narrowband wavelength-selective extraction and intensity tuning in on-chip nonlocal metasurfaces, we fabricated the designed sample using plasma-enhanced chemical vapor deposition (PECVD) and conventional electron-beam lithography (EBL). Detailed fabrication steps are provided in the Methods section. Figure 3b exhibits the SEM image of the fabricated on-chip sample with *θ* varying from 0° to 20°. The broadband polarized laser (500–800 nm) is coupled into the waveguide via an end-fire manner utilizing a fiber collimator. More details on the experimental measurements can be found in Supplementary Sections S11–S13, Supplementary Information. For a fixed scaling factor (*S* = 1), as the asymmetry parameter increases, Fig. 3c exhibits a concomitant rise in the extraction intensity, thereby experimentally verifying the continuous intensity tuning by introducing the asymmetry parameter. Notably, the spectral values were obtained by normalizing the extraction intensity at each wavelength with the incident light source intensity, reflecting relative extraction intensities. On the other hand, we further fabricated the on-chip sample with the scaling factor *S* varying from 0.88 to 1 (keeping a fixed tilting angle *θ* = 25°), as shown in the SEM images in Fig. 3d. As the scaling factor increases, the measured extracted peak position in Fig. 3e redshifts from 570 nm to 630 nm, which is in good agreement with the varying trend in theory. More details about the comparison of simulated and experimental extraction spectra can be found in Supplementary Sections S14 and S15, Supplementary Information. In addition, more details on simulation analysis of the fabrication-induced structural offset and its influence on spectral characteristics, and the origin and characteristics of the secondary peak are provided in Supplementary Section S16, Supplementary Information.

In addition, we further numerically investigated the extraction spectra of q-BIC-assisted periodic arrays of *M* × *N*-unit cells with *N* varying from 1 to 50. Here, *M* and *N* are the row sequence number (*y*-direction) and column sequence number (*x*-direction) of the arrays, respectively, where *M* approaches infinity due to the periodic boundary conditions in the *y*-direction. The spectra (Fig. 3f) and maximum extraction efficiency (Fig. 3g) of these arrays showcase a continuous increase in peak extraction with the expansion of the array size *N* along the *x*-direction. Note that after *N* > 20, the extraction efficiency peak reaches saturation (~0.5) and does not increase substantially even if the array size increases. More details on the analysis of guided wave propagation and energy flow can be found in Supplementary Section S17, Supplementary Information.

**Fig. 4:**
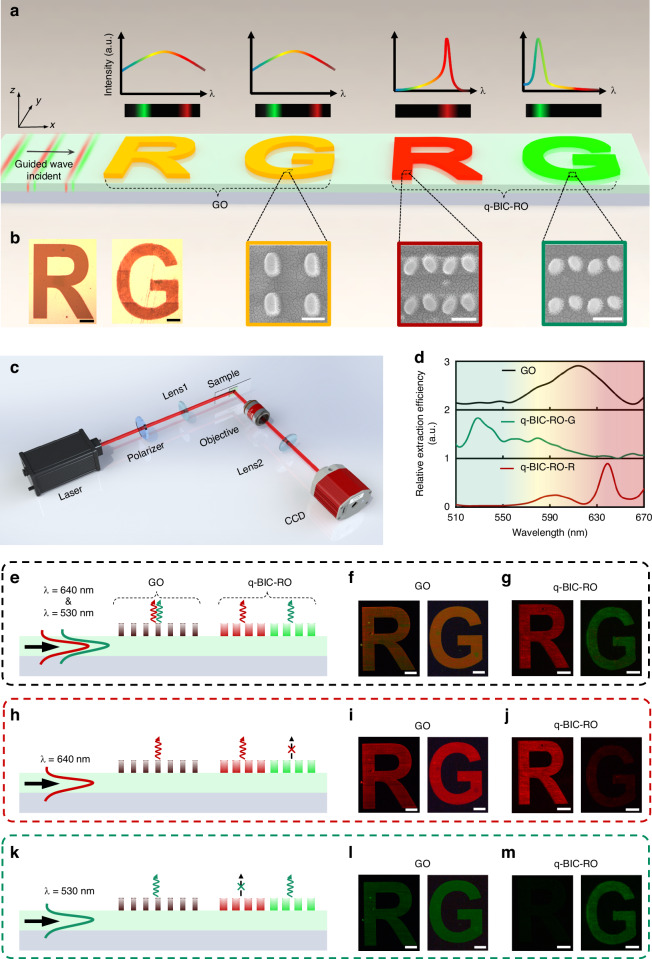
Experimental characterization of color routing microimage display based on on-chip q-BIC-assisted metasurface. **a** Conceptual illustration of color routing display functionality based on the on-chip q-BIC-assisted metasurface. Bottom: SEM image of the fabricated patterns. Scale bar: 250 nm. **b** Optical photograph of the fabricated “R” and “G” letters composed of q-BIC-assisted meta-diatom patterns. Scale bar: 30 μm. **c** Experimental setup to characterize the on-chip near-field microimages. **d** Measured extracted spectral profile comparison between the traditional grating out-coupler (GO) and the proposed on-chip q-BIC-assisted meta-router (q-BIC-RO). q-BIC-RO-R: q-BIC-assisted red meta-router; q-BIC-RO-G: q-BIC-assisted green meta-router. **e**, **h**, **k** Side view showing the functionality of the q-BIC-assisted metasurface for color routing. **f**, **g** Optical microscope images of “R” and “G” letters based on the traditional GO and the proposed on-chip q-BIC-RO design when guided waves with wavelengths of 640 nm and 530 nm are simultaneously propagating from the *x*-direction. Scale bar: 30 μm. **i**, **j** Optical microscope images of “R” and “G” letters, taken at the wavelength of 640 nm. Scale bar: 30 μm. **l**, **m** Optical microscope images of “R” and “G” letters, taken at the wavelength of 530 nm. Scale bar: 30 μm

To further explore the selective extraction and routing capabilities, we demonstrate its potential applications in color routing for microimage displays. Figure 4a schematically compares a conventional on-chip grating out-coupler (GO) with the proposed q-BIC-assisted meta-router (q-BIC-RO), highlighting the shift from broadband out-coupling to narrowband, wavelength-selective extraction. Using our designed on-chip q-BIC-assisted meta-diatom array, we fabricated solid-color fonts forming the letters “R” and “G” (Fig. 4b), alongside traditional GO-formed “R” and “G” letters for comparison, as shown in the corresponding SEM images at the bottom of Fig. 4a.

To evaluate the on-chip color routing display performance, we used an optical microscope with a 50× objective lens and a CCD camera to focus near the sample surface and capture the extracted light intensity distributions from the on-chip pattern (Fig. 4c). More details on the measurements can be found in the Methods section. The experimental extraction spectra for the conventional GO, and the q-BIC-assisted green and red ROs, are shown in Fig. 4d, confirming that the q-BIC-RO achieves narrowband (~20 nm) wavelength-selective extraction, in contrast to the broadband (~60 nm) out-coupling of the conventional GO. The GO, composed of single meta-atom pixels, was deliberately used as a comparison, as its extraction intensity lacks contrast at the red and green wavelengths. More details on the comparison of simulated extracted spectra of the on-chip q-BIC-RO with conventional GO, as well as the GO selection, are provided in Supplementary Section S18, Supplementary Information.

To further illustrate the practical selective extraction and routing capabilities of the on-chip q-BIC-assisted metasurface, Fig. 4e, h, k schematically shows the extraction performance of conventional GO and q-BIC-ROs under guided waves with different wavelength combinations. When red (640 nm) and green (530 nm) guided waves are incident (Fig. 4e), the conventional broadband GO extracts both wavelengths simultaneously without notable contrast, resulting in the letters “R” and “G” appearing yellow due to the mixture of red and green (Fig. 4f). In contrast, the q-BIC-ROs extract only the red and green wavelengths separately, showing distinct red and green colors, respectively (Fig. 4g). Similarly, when only red (640 nm) (Fig. 4h) or green (530 nm) (Fig. 4k) guided waves are incident, the conventional GO extracts the corresponding colors without selectivity (Fig. 4i, l), while the q-BIC-ROs achieve selective extraction of red (Fig. 4j) and green (Fig. 4m), confirming successful color routing in the microimage display. It is worth noting that the relative extraction efficiency ratio of pattern “R” (31.76) at 530 nm and 640 nm surpasses that of pattern “G” (19.31), thereby demonstrating a more pronounced color filtering effect, as illustrated in Fig. 4j, m. More details can be found in Supplementary Section S19, Supplementary Information.

**Fig. 5:**
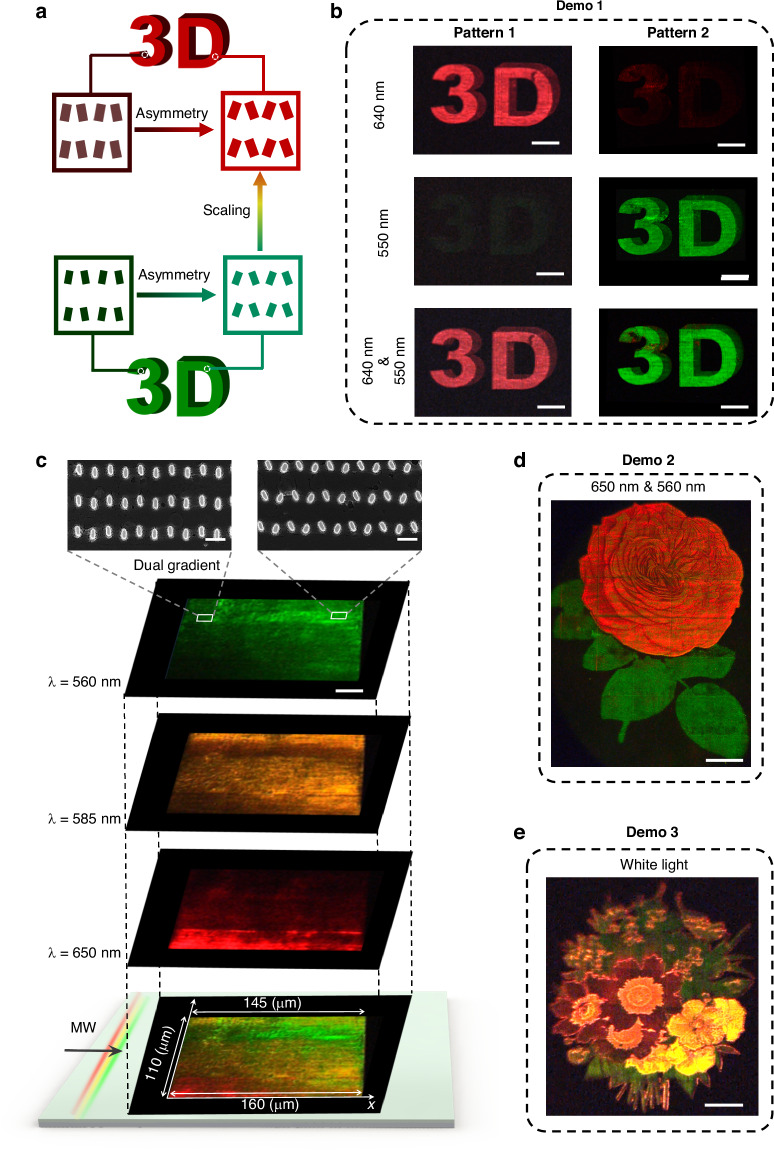
Experimental demonstration of multicolor image meta-display based on on-chip q-BIC-assisted color pixels. **a** Schematic diagram of designing stereoscopic and multicolor nano-printing metasurface patterns to achieve simultaneous color and brightness tuning. **b** Demo 1: Captured optical images of stereoscopic “3D”. Scale bar: 30 μm. **c** Optical images of the on-chip dual-gradient metasurface taken at different wavelengths. MW: Multiwavelength. Top: SEM image of the fabricated dual-gradient on-chip metasurface sample. Scale bar: 250 nm. The corresponding parameters are *L*_0_ = 100 nm, *W*_0_ = 60 nm, and *P*_0_ = 400 nm when *S* = 1. **d** Demo 2: Experimental optical dual-color grayscale image “Red Flower & Green Leaf”. Scale bar: 50 μm. **e** Demo 3: Experimental optical multicolor grayscale image “Flowers & Leaves” under white light incidence. Scale bar: 50 μm

Next, by encoding color and brightness information into different meta-diatom pairs with varying geometrical dimensions and tilting angles, we can create on-chip stereoscopic and multicolor nano-printing images. A stereoscopic “3D” pattern was chosen as the target image. Using a parameter-matching approach (Fig. 5a), the image was pixelated, segmented into different color and intensity channels, and each pixel was matched to the closest color and intensity achievable by the BIC (or q-BIC)-assisted meta-diatom array. In Demo 1, two distinct patterns, Pattern 1 and Pattern 2, were fabricated to depict red and green stereoscopic “3D” images, as shown in Fig. 5b. This highlights the differences in extraction color and intensity between q-BIC-assisted meta-diatoms at varying tilting angles and scaling factors.

Additionally, we fabricated an on-chip dual-gradient metasurface, with unit cell scaling continuously varying along the *x*-direction (*S* = 0.8–1) and the meta-diatom tilting angle changing along the *y*-direction (0°–22°), as shown in the SEM image in Fig. 5c. The optical performance of this dual-gradient metasurface was demonstrated under single-wavelength (560 nm, 585 nm, and 650 nm) and multi-wavelength (MW) extraction, showing spectral selectivity and continuous intensity tuning. Leveraging this dual-gradient metasurface, we demonstrated two grayscale images: “Red Flower & Green Leaf” in Demo 2 (Fig. 5d) under dual-wavelength incidence (650 nm and 560 nm), and the multicolor grayscale image “Flowers & Leaves” in Demo 3 (Fig. 5e) under white light. The experimental photograph of “Red Flower & Green Leaf” captured under the white light source incidence is provided in Supplementary Section S20, Supplementary Information. More details on the effect of array size on the spatial mapping of color pixels of complex and detailed patterns can be found in Supplementary Sections S21 and S22, Supplementary Information. Both images display vibrant colors and smooth brightness transitions. It is worth noting that under broadband (white-light) illumination, the Q-factor-dependent amplitude modulation inevitably causes spectral broadening, which could affect the saturation of the extracted color. More detailed analysis of the relationship between the Q-factor, amplitude modulation, and the impact on color saturation of the extracted light can be found in Supplementary Section S23, Supplementary Information. Importantly, thanks to the on-chip propagation scheme, the captured images are free from zeroth-order background interference, and no additional optical elements such as polarization analyzers, were required.

## Discussion

In summary, we propose and experimentally demonstrate nonlocal on-chip metasurface color routers patterned on a waveguide utilizing symmetry-broken q-BICs. By elaborately engineering the geometric asymmetry and scaling factor of the meta-diatom pairs at the nanoscale, we achieve simultaneous modulation of extraction intensity and primary wavelength of the out-coupling lightwave, resulting in narrowband extraction spectra with minimal background noise. As a proof of concept, we present several multicolor routing demonstrations that validate the ability to guide and route individual wavelengths into free space through spatial mapping and cascading distinct meta-diatom color pixels. Notably, on-chip q-BIC-assisted color routers are cascade multiplexing with a marked improvement in EUE performance beyond conventional designs. Additionally, the presented multicolor meta-displays benefit from the absence of zeroth-order background interference due to the on-chip propagation scheme. Overall, the demonstrated narrowband spectral extraction provides critical advantages for on-chip photonic systems, enabling crosstalk-free color-multiplexed displays and high-selectivity guided-wave routing in WDM architectures. We envision that such an on-chip nonlocal meta-optics platform with the merit of miniaturization and integration, holds promise for diverse applications in next-generation wearable meta-display devices, multiplexing information routing, and intelligent integrated photonic systems.

## Materials and methods

### Sample fabrication

The fabrication of the on-chip BIC (or q-BIC)-assisted meta-diatom array commenced with the deposition of a 220-nm-thick Si₃N₄ waveguide layer on a 500-μm-thick fused silica substrate using plasma-enhanced chemical vapor deposition (PECVD). This was followed by the deposition of a 380-nm-thick *α*-Si layer. A 120-nm-thick layer of polymethyl methacrylate (PMMA) was subsequently spin-coated onto the *α*-Si surface and baked at 150 °C for 180 s. To mitigate charge accumulation during lithography, a conductive polymer was spin-coated on top of the PMMA layer. Next, the on-chip BIC (or q-BIC)-assisted meta-diatom array was patterned using electron beam lithography (EBL, Raith eLINE Plus, 20 kV), followed by development in a developer solution for 80 s. A 20-nm-thick chromium (Cr) layer was then deposited via thermal evaporation to serve as a dry-etch mask. The PMMA resist was removed through a lift-off process using acetone, leaving the Cr mask in place. The Cr patterns were transferred to the *α*-Si layer through the reactive ion beam etcher (RIE-150A, Tailong Electronics) machine and a gas mixture of SF_6_/CHF_3_/O_2_, and the remaining Cr mask was finally removed with a Cr etchant.

### Numerical simulation

The finite-difference time-domain (FDTD) method was employed to implement numerical simulations. We established periodically distributed *α*-Si meta-diatom pairs above the Si_3_N_4_ (*n* = ~2.05) waveguide with a SiO_2_ substrate (*n* = 1.45) to calculate electric-field intensity |*E*|^2^ distributions (Fig. 2c–e, j–l) and the extraction spectra (Figs. 2f–i and 3f, g) of the extracted light. Here, we applied perfectly matched layers (PML) conditions along the *x*- and *z*-directions, and periodic boundary conditions along the *y*-direction to perform the three-dimensional simulations. The propagating guided waves are set as the transverse electric mode (TE_0_) in the slab waveguide.

### Optical measurement

To characterize the optical extraction performance of on-chip BIC (or q-BIC)-assisted metasurfaces, we utilized the supercontinuum laser (SC-PRO, covering the spectral range of 430–2400 nm) to generate broadband polarized light sources (500–800 nm) that were coupled into the waveguide in an end-fire manner by a fiber collimator (F240SMA-532, Fiber Collimation Package, *λ* = 532 nm, *f* = 7.87 mm). The extracted light was collected by a 100× objective (NA = 0.90), and the corresponding wavelength-resolved spectra were obtained at the Fourier back focal plane through an angle-resolved microscopic spectrometer (ARMS, Ideaoptics Inc.). The experimental near-field multicolor images were collected and captured by employing a microscope with a 50× (NA = 0.80) objective and a CCD camera.

## Supplementary information

Supplementary Information for On-Chip Nonlocal Metasurface for Color Router: Conquering Efficiency-Loss from Spatial-Multiplexing

## Data Availability

The data that support the findings of this study are available within the paper and the supplementary. Additional data related to this paper are available from the corresponding authors upon reasonable request.
