# Supplementary material for: On-chip nonlocal metasurface for color router: conquering efficiency-loss from spatial-multiplexing
Source: Light Sci Appl. 2026 Jan 12;15:66. doi: 10.1038/s41377-025-02146-9 (PMC12791144; doi:10.1038/s41377-025-02146-9)
Supplement: Supplementary file 1 — Supplementary Information for On-Chip Nonlocal Metasurface for Color Router: Conquering Efficiency-Loss from Spatial-Multiplexing [file 41377_2025_2146_MOESM1_ESM.pdf]

Supplementary Information for

On-Chip Nonlocal Metasurface for Color Router: Conquering Efficiency-Loss from Spatial-Multiplexing

Yangyang Shi[1], Shuai Wan[1], Zejing Wang[1], Runlong Rao[1], Zhongyang Li[1,2,*]

Y. Shi, S. Wan, Z. Wang, R. Rao, Prof. Z. Li
[1]Electronic Information School, Wuhan University, Wuhan 430072, China

Prof. Z. Li
[2]Wuhan Institute of Quantum Technology, Wuhan 430206, China

*Corresponding author. Email: *zhongyangli@whu.edu.cn* (Z. L.)

S1. Recent advances in on-chip metasurfaces and their comparison

Table S1. Comparison between our work and other on-chip metasurfaces for guided wave manipulation.

Work	Optical parameter control	Modulation strategy or mechanism	Principle optical functionalities	Wavelength controllability
Ref. 5	Phase only	Resonant phase	Beam-steering, focusing, OAM	NO
Ref. 11	Binary phase/amplitude	Detour phase	OAM	NO
Ref. 13	Phase and polarization	Geometric phase	Higher-order Poincaré sphere beams	NO
Ref. 14	Phase only	Resonant phase	Focusing, fresnel hologram	NO
Ref. 15	Amplitude, phase, and polarization	Resonant phase and geometric phase	Focusing, fresnel hologram, Poincaré beams	NO
Ref. 16	Phase only	Detour phase and geometric phase	Hologram, AR display	NO
Ref. 18	Phase only	Detour phase	Fresnel hologram	NO
Ref. 20	Phase and binary amplitude	Detour phase and meta-diatom interference	Hologram, transparent screen display, AR display	NO
Ref. 22	Continuous amplitude	Meta-diatom interference	Continuous grayscale image display	NO
Ref. 23	Phase only	Geometric phase	Focusing, fresnel hologram	NO
Ref. 26	Phase, polarization, and amplitude	Detour phase, geometric phase, and resonant phase	Nano-printing and hologram	NO
Ref. 30	Phase and polarization	Geometric phase	Beam-steering, focusing	NO
**Our work**	**Wavelength and amplitude**	**Quasi-bound state in the continuum**	**Color routing and multicolor meta-display**	**YES**

Table S1 provides a comprehensive incorporation and comparison of our on-chip metasurface work with recent advancements for manipulating the extracted guided

waves. To date, on-chip metasurfaces have been developed to control the amplitude, phase, and polarization of the extracted lightwave, enabling versatile optical functionalities, including on-chip beam steering and focusing, orbital angular momentum (OAM) beams, nano-printing, meta-hologram, and screen display, etc. Despite various modulation strategies and mechanisms for modulating phase, amplitude, and polarization having been extensively explored, the challenge of achieving wavelength-controllable extraction with on-chip metasurfaces remains unresolved.

Typically, most on-chip metasurfaces exhibit a broadband optical response with minimal wavelength selectivity, providing similar extraction functionality across various wavelengths, as demonstrated in our previous work [Ref. 16]. In that study, we showcased on-chip broadband beam deflection performance (Fig. 1c of Ref. 16) and broadband on-chip holographic display (Supporting Information of Ref. 16). Here, to further illustrate the broadband response of conventional meta-atoms, we simulated a periodic distributed on-chip meta-atom array for extracting guided waves, as depicted in Fig. S1a. Figure S1b plots the far-field intensity $|E|^2$ as a function of the deflection angle and wavelength of the extracted light. It reveals that broadband guided waves in the range of 570 nm to 800 nm are effectively out-coupled into free space, achieving an average extraction efficiency of approximately 0.35 (Fig. S1c).

The ability to modulate wavelength while selectively controlling extraction on an on-

chip platform is essential for advancing integrated photonic applications, such as wavelength-division multiplexing (WDM) and color routing. Specifically, such narrowband response based on our quasi-bound states in the continuum (q-BIC)-based design is highly beneficial for color-multiplexed image meta-displays, where spectral overlap can result in crosstalk and degraded color fidelity. Beyond display applications, our design provides a practical solution for WDM-based guided wave routing, enabling selective extraction or redirection of different spectral components within compact on-chip architectures. This is particularly useful for signal demultiplexing, optical interconnects, and programmable photonic processors. More importantly, beyond wavelength selectivity alone, our platform enables simultaneous and flexible modulation of both the spectral position and the extraction intensity of guided waves—an ability not achieved in previous works, and providing a robust toolkit for future nanophotonic integration. We believe this work represents a significant advancement in the state-of-the-art of on-chip metasurfaces for guided wave manipulation and opens new frontiers in the spectral and spatial control of light at the subwavelength scale. Therefore, our work represents a significant advancement in the state-of-the-art on-chip metasurfaces for guided wave manipulation.

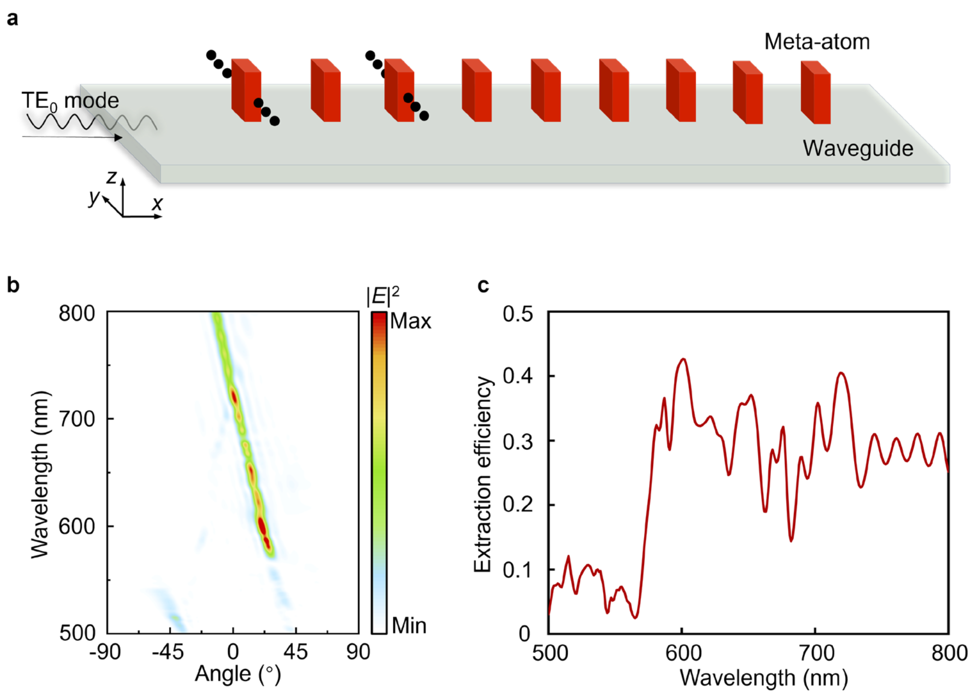

Fig. S1 (a) Schematic of a conventional periodically distributed on-chip meta-atom array for extracting guided waves. The corresponding parameters of the meta-atom are L = 180 nm, W = 100 nm, P_x = 400 nm, P_y = 300 nm, and H = 380 nm. (b) Simulated far-field intensity $|E|^2$ as a function of deflection angles (x-axis) and wavelength (y-axis). (c) Simulated corresponding extracted spectrum at the broadband visible range.

S2. Comparison and discussion of energy utilization efficiency (EUE) from on-chip cascading metasurface and free-space spatial multiplexing metasurface

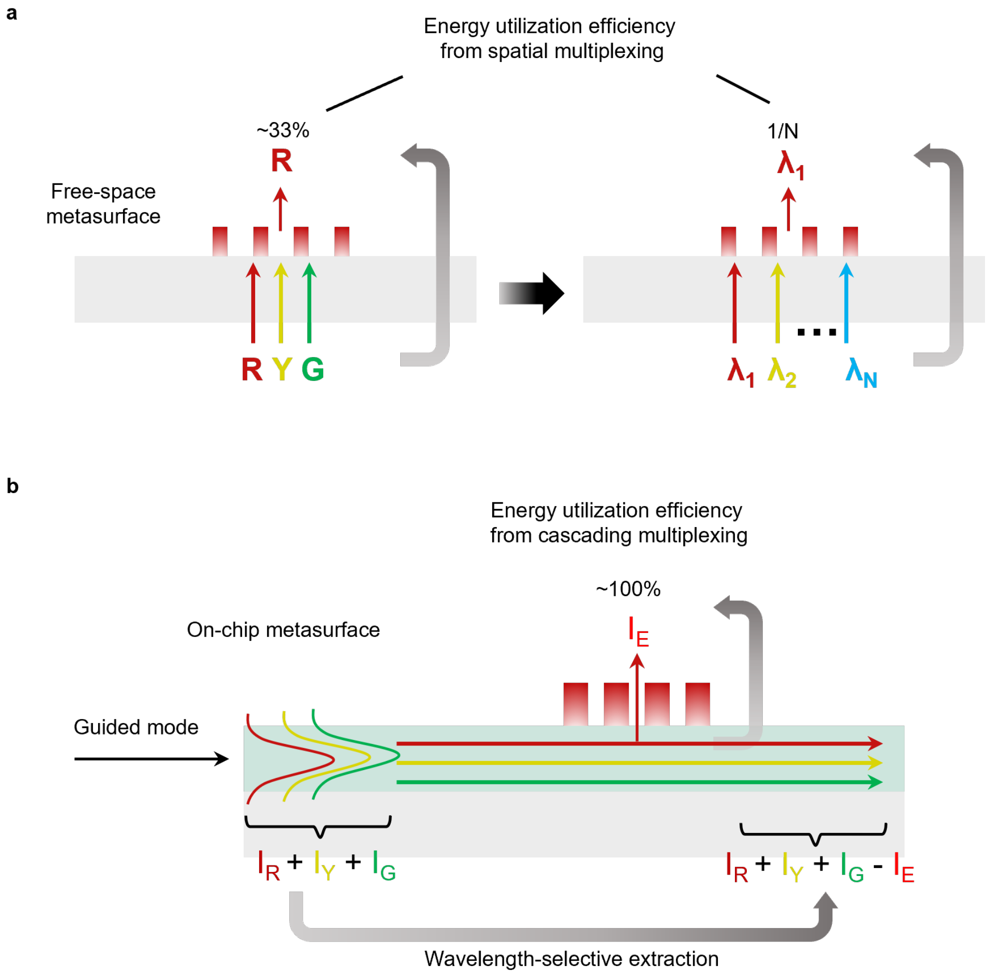

Fig. S2 (a) Schematic of energy utilization efficiency of free-space metasurface for color routing based on spatial multiplexing. (b) Schematic of energy utilization efficiency of waveguide-integrated metasurface for color routing based on on-chip cascading multiplexing.

Conventional free-space metasurfaces for color routing or filtering, based on spatial multiplexing, typically allow only a single target wavelength to pass through, resulting in the loss of other wavelengths due to absorption or reflection outside the filter's passband. As shown in Fig. S2a, when illuminated with three color channels (red, yellow, and green), it allows only the red wavelength to pass through, discarding the yellow and green components. We defined free-space energy utilization efficiency

(EUE) as the ratio of the transmitted light intensity of a single-wavelength channel to the total incident light intensity, which is inherently constrained to an upper limit of ~ 33% under the assumption of negligible optical losses. When extended to N-wavelength incident channels, the maximum EUE is limited to $1/N$. Although various approaches have been employed to enhance the efficiency of free-space metasurface color routers, including volumetric meta-optics, dispersion engineering, and inverse design, these devices encounter challenges related to complex structure optimization, low degrees of design freedom, and fabrication difficulties.

Beyond free-space metasurfaces, on-chip metasurfaces present distinct advantages by enabling the horizontal cascading of multiple arrays. This cascading multiplexing strategy, facilitated by the on-chip propagation scheme, promises improved energy allocation compared to free-space spatial multiplexing. As illustrated in Fig. S2b, when broadband guided light interacts with a red-color periodic q-BIC-assisted array, it selectively extracts and routes guided waves, while allowing light waves of other colors (e.g., yellow and green) to continue propagating without energy loss. The initial total intensity can be expressed as $I_T = I_R + I_Y + I_G$ (for three color channels), while the intensity extracted upon passing through the array is denoted as I_E. The intensity that continues to propagate in the waveguide after passing through the array is recorded as $I_C = I_R + I_Y + I_G - I_E$, assuming minimal propagation losses and optical absorption losses. Consequently, the EUE from on-chip cascading multiplexing, calculated as $I_E/(I_T - I_C)$, approaches unity. Therefore, such an on-chip cascading multiplexing design could

overcome the EUE limitation inherent in free-space spatial multiplexing, facilitating advancement in integrated photonic applications such as WDM and color routing.

It is important to note that extraction efficiency and EUE are completely different concepts. The extraction efficiency is defined as the ratio of the light intensity (I_E) extracted into free space to the total incident light intensity (I_T), indicating the performance of the on-chip metasurface to extract guided light. Nevertheless, the EUE represents the utilization efficiency of the propagating guided light in the waveguide. Therefore, our improvement is achieved relative to conventional spatial-multiplexed metasurface-based color routers in free space—a conceptual advance that, to our knowledge, has not been previously reported in on-chip light manipulation studies. Importantly, this does not imply an improvement in absolute extraction efficiency compared to state-of-the-art on-chip outcoupling metasurfaces. Nevertheless, we believe that further improving the overall extraction efficiency could in turn enhance EUE, and we envision this as a promising direction for future work. For instance, combining inverse design and topology optimization strategies may enable the simultaneous realization of both high spectral selectivity and efficient light outcoupling.

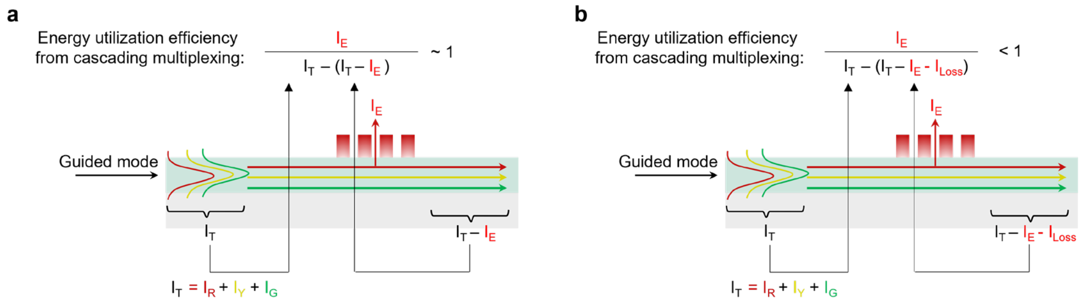

Fig. S3 (a) Schematic diagram of EUE calculation of waveguide-integrated metasurface color routing based on on-chip cascade multiplexing without considering optical losses. (b) Schematic of EUE calculation based on on-chip cascading multiplexing under considering optical losses. I_T: the total incident light intensity; I_E: the extracted light intensity; I_{Loss}: optical loss intensity (including the propagation and optical absorption losses).

Here, we would like to point out that the EUE close to the unity we claimed was based on the assumption of minimal propagation loss and optical absorption losses. As shown in Fig. S3a, assuming no optical losses are considered, EUE is theoretically calculated to be close to unity.

When assuming that optical losses including propagation and material absorption (from the intrinsic loss of the materials and the enhanced loss due to the structure resonance) are taken into account, as illustrated in Fig. S3b, EUE is theoretically calculated to be less than 1. Specifically, the initial total intensity can be expressed as $I_T = I_R + I_Y + I_G$ (for three color channels), while the intensity extracted upon passing through the array is denoted as I_E. The intensity that continues to propagate in the waveguide after passing through the array is recorded as $I_C = I_T - I_E - I_{Loss}$, where I_{Loss} represents the optical losses (including propagation and material absorption losses). Consequently, the EUE from on-chip cascading multiplexing, calculated as $I_E/(I_T - I_C)$, is less than 1.

Although the EUE value could be lower (<1) due to the optical loss, we would like to point out that the EUE from our on-chip propagation and cascaded platform could be higher compared to free space alternative (It is constrained an upper limit of ~ 33% for three color channels under the assumption of negligible optical losses, and when

extended to *N*-wavelength incident channels, the maximum EUE is limited to 1/N). Here, to provide more direct evidence for the high EUE value, we numerically simulated and investigated the EUE of the on-chip periodic meta-diatom array, as shown in Fig. S4a-c below. Specifically, the meta-diatom arrays consist of 14 × *N*-unit cells, where *N*—the row sequence number (*y*-direction) of the array approaches infinity due to the periodic boundary conditions in the *y*-direction, and the column sequence number (*x*-direction) is 14. In order to evaluate the EUE value in the simulation, we strategically set two field monitors positioned in the end port (Monitor 1) of the waveguide and the free-space region (Monitor 2) above the metasurface to record the corresponding energy flow and intensity, as illustrated in Fig. S4g. Meanwhile, we removed the metasurface to simulate the guided wave propagation (Fig. S4d-f) and set the same field monitor (Monitor 3) to record the output light intensity at the waveguide end (Fig. S4h). Then, we chose three input wavelengths of 557 nm, 596 nm, and 652 nm to simulate the three-wavelength channel case and to obtain the corresponding light intensity T_1, T_2, and T_3 at the extraction spectral peak wavelength of 650 nm. Finally, according to the formula of EUE = $T_2/(T_3 - T_1)$, the calculated EUE value from the simulation is approximately 0.62, which is nearly twice the theoretical upper limit of ~ 33% for three color channels in the free space alternative. Therefore, achieving an EUE value of near unity in practical implementations may be challenging. Such an ideal value (approaching ~1) can potentially be achieved under the assumption of minimal propagation losses and optical absorption losses. Nevertheless, despite the presence of these unavoidable losses, our proposed on-chip platform still demonstrates a

significantly higher EUE compared to conventional free-space approaches.

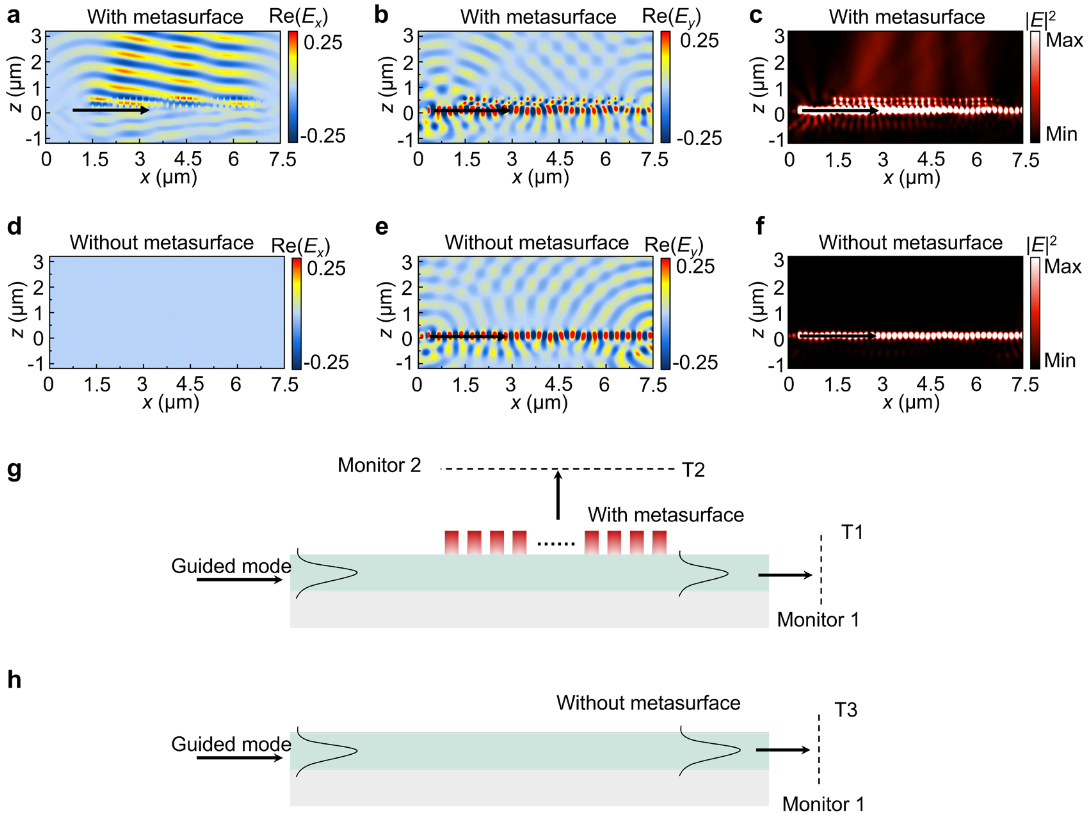

Fig. S4 Simulated extraction of guided waves by the metasurface integrated onto the waveguide. (a-c) Calculated corresponding electric-field E_x, electric-field E_y, and total field intensity $|E|^2$ profiles at the wavelength of 650 nm with metasurfaces integrated onto the waveguide. (d-f) Electric-field E_x, E_y, and intensity $|E|^2$ profiles, for the waveguide without metasurface integration. The TE_0 mode guided wave is incident from the x direction. The propagation direction of the guided waves is denoted by the black arrows. The corresponding parameters are $L = 130$ nm, $W = 65$ nm, $P_x = P_y = 400$ nm, and $H = 380$ nm. (g-h) Schematic of calculating the EUE value in the simulation.

S3. The refractive index and extinction coefficient of *α*-Si in the simulation

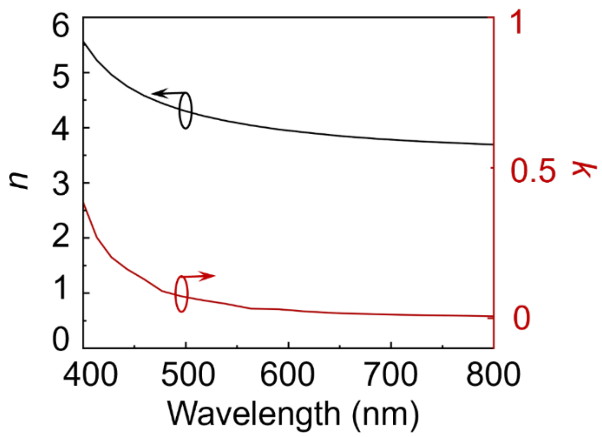

Fig. S5 The real part *n* and imaginary part *k* of refractive indexes of Si materials in the simulation.

Regarding the refractive index and extinction coefficient of *α*-Si utilized in our simulations, we have plotted the refractive index distribution of Si material at different wavelengths in the simulation, as shown in Fig. S5.

S4. The on-chip q-BIC-assisted meta-diatom design from the perturbed lattice

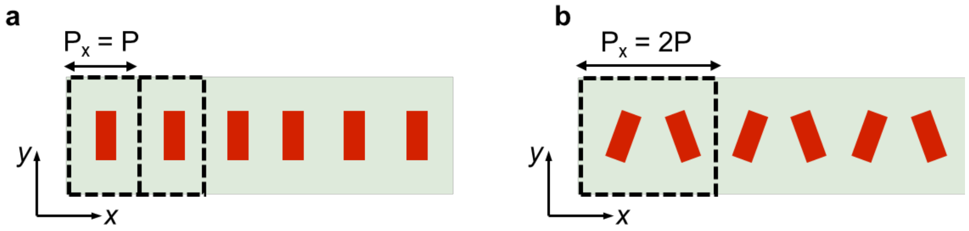

Fig. S6 (a) Schematic top-view of an on-chip meta-atom array composed of a single nanoblock pixel with a period of $P_x = P$. (b) Schematic top-view of an on-chip q-BIC-assisted meta-diatom array composed of two tilted nanoblocks with a period of $P_x = 2P$.

Here, the key realization to enable an on-chip meta-diatom array with wavelength-selective extraction is that we can revoke the symmetry protection that suppresses guided wave radiation by introducing a period-doubling symmetry-breaking perturbation to the unit cell. As shown in Fig. S6a, when the tilting angle θ of the meta-diatom pair is 0°, it can be regarded as an array with a period of $P_x = P$, marked by the dotted outline. Due to the decreasing period, light extracted from the propagating guided waves gains a transverse wave vector so large that it exceeds the maximum supportable wave number in free space, and thus it is bounded in the waveguide. In this case, the structure supports a symmetry-protected BIC and is in an unperturbed state. Such a BIC is unstable against perturbations that break the in-plane inversion symmetry, and it would be transformed into a quasi-BIC with a finite Q factor. As shown in Fig. S6b, when the tilting angle $\theta > 0°$, the meta-diatom pair is perturbed, and the array period along the x-direction is artificially doubled. Then, the guided mode is extracted into free space, and the bound wave mode is transformed into a leaky q-BIC mode, which can be tailored to exhibit narrowband spectral features due to the sharp, high-Q-

factor resonance response at specific frequencies. In our work, we have neglected the gradual decrease of q-BIC power density as the guided waves propagate along the waveguide, which would not cause notable issues for weak perturbations due to the low leakage rates.

S5. Numerical simulations of the far-field intensity profile of on-chip q-BIC-assisted arrays with dimensional variation

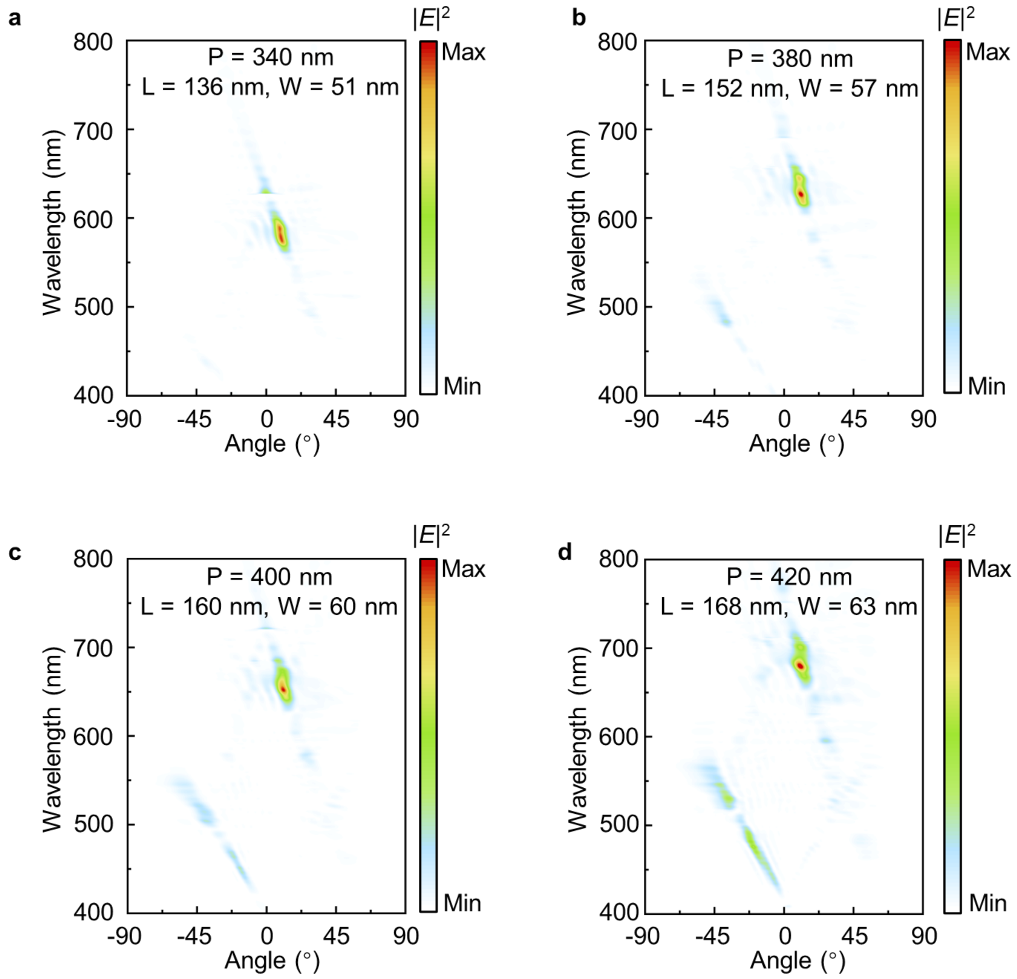

Fig. S7 (a-d) Simulated far-field intensity $|E|^2$ as a function of deflection angles (x-axis) and wavelength (y-axis) for on-chip q-BIC-assisted arrays with distinct structural parameters.

Figures S7a-d respectively plot the far-field intensity $|E|^2$ as a function of the deflection angle and wavelength of the out-coupling light for several on-chip q-BIC-assisted arrays with different scaling factors. It is observed that on-chip q-BIC-assisted meta-diatom arrays can selectively extract narrowband guided waves into free space, which is significantly distinct from the conventional meta-atom array with broadband extraction performance shown in Fig. S1b-c.

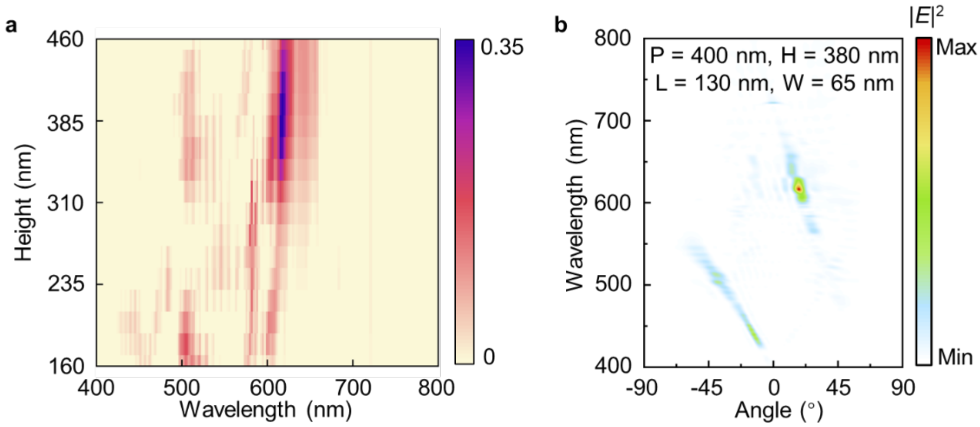

Fig. S8 The effect of meta-diatom height on on-chip wavelength-selective extraction. (a) Simulated extraction spectra corresponding to distinct q-BIC-assisted meta-diatom arrays with heights varying from 160 nm to 460 nm. (b) Simulated far-field intensity $|E|^2$ as a function of deflection angles (x-axis) and wavelength (y-axis). The corresponding parameters are L = 130 nm, W = 65 nm, $P_x = P_y$ = 400 nm, and H = 380 nm.

Here, we numerically studied the extraction spectra for on-chip q-BIC-assisted Si meta-diatom arrays with varying heights from 160 nm to 460 nm at the broadband visible range, as shown in Fig. S8a. When the height approaches ~ 380 nm (Fig. S8b), the peak of the extraction efficiency reaches a maximum of ~ 0.35, while exhibiting a sharp spectral extraction.

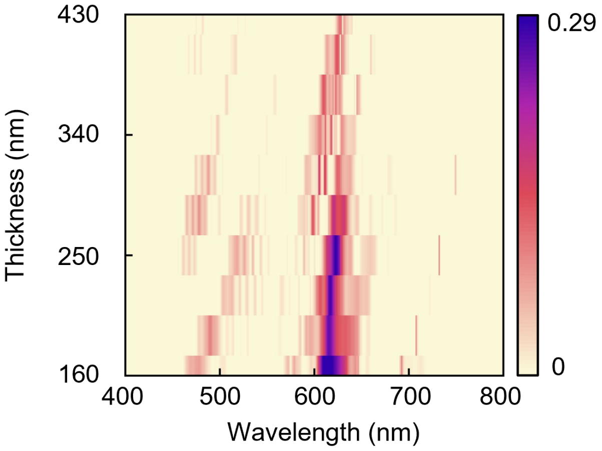

Fig. S9 Simulated extraction spectra of the q-BIC-assisted meta-diatom array integrated on waveguides with distinct thicknesses, ranging from 160 nm to 430 nm. The corresponding parameters are L_0 = 160 nm, W_0 = 60 nm, and P_0 = 400 nm.

We further numerically studied the extraction spectra of the meta-diatom array integrated on waveguides with distinct thicknesses, ranging from 160 nm to 430 nm, as shown in Fig. S9. It can be observed that, compared to our original waveguide design with a thickness of 220 nm, the peak wavelength of the extracted spectrum exhibits a shift of only ±3 nm within a thickness variation range of 220 ± 30 nm (i.e., from 190 nm to 250 nm).

S6. Numerical simulations of on-chip q-BIC-assisted metasurfaces for color routing and their comparison with conventional on-chip grating out-couplers

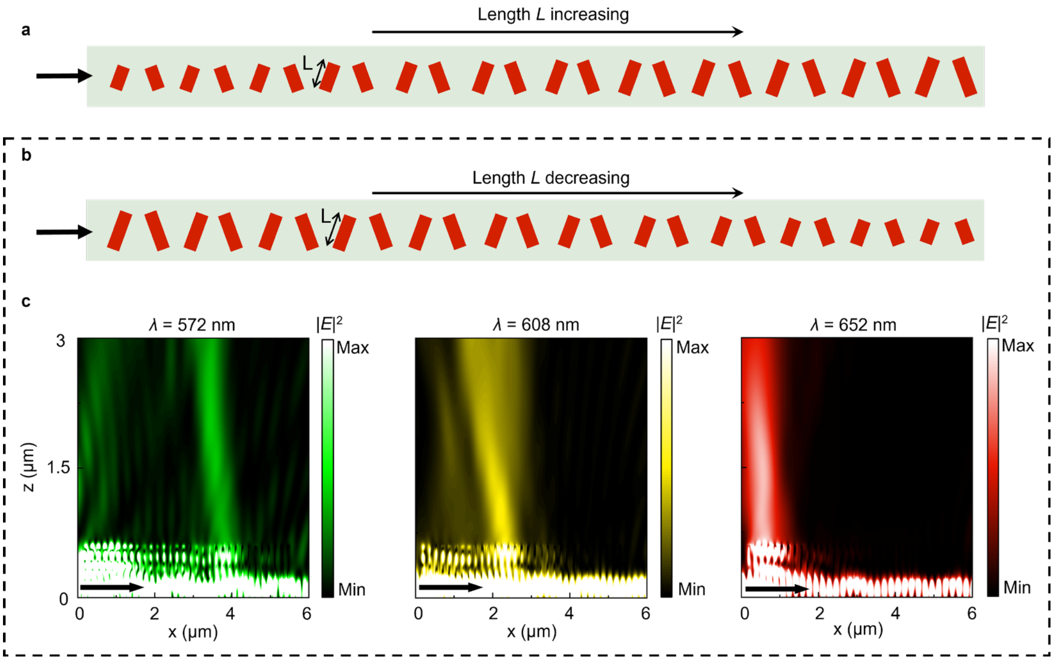

Fig. S10 Numerical simulations of on-chip q-BIC-assisted metasurfaces for color routing. (a) Schematic of an on-chip q-BIC-assisted continuous gradient array with the meta-diatom (tilting angle θ = 20°) length L increasing from 95 nm to 170 nm (keeping the width fixed at 60 nm) along the x-direction. (b) Schematic of an on-chip q-BIC-assisted continuous gradient array with the meta-diatom length L decreasing from 170 nm to 95 nm (keeping the width fixed at 60 nm). (c) Simulated electric-field intensity ($|E|^2$) profiles extracted by the gradient array in (b) from the waveguide at the wavelengths of 572 nm, 608 nm, and 652 nm. The black arrows represent the propagation direction of the guided waves.

Figure S10a shows the schematic of an on-chip q-BIC-assisted continuous gradient array with the meta-diatom (tilting angle θ = 20°) length L increasing from 95 nm to 170 nm (keeping the width fixed at 60 nm) along the x-direction. The corresponding simulated electric-field intensity $|E|^2$ profiles at x-z cross-section have been exhibited in Fig. 2j-l in the main text. To further illustrate the color routing performance, we arrange the array in Fig. S10a in an opposite gradient of length L decreasing from 170 nm to 95 nm, as shown in Fig. S10b. The simulated electric-field intensity $|E|^2$ profiles at x-z cross-section (Fig. S10c) also exhibit that guided waves with different wavelength

components are strongly extracted into free space from respective different locations. The significant difference is that due to the opposite dimensional gradient arrangement, lightwaves of the same wavelength are extracted from opposite positions of the array.

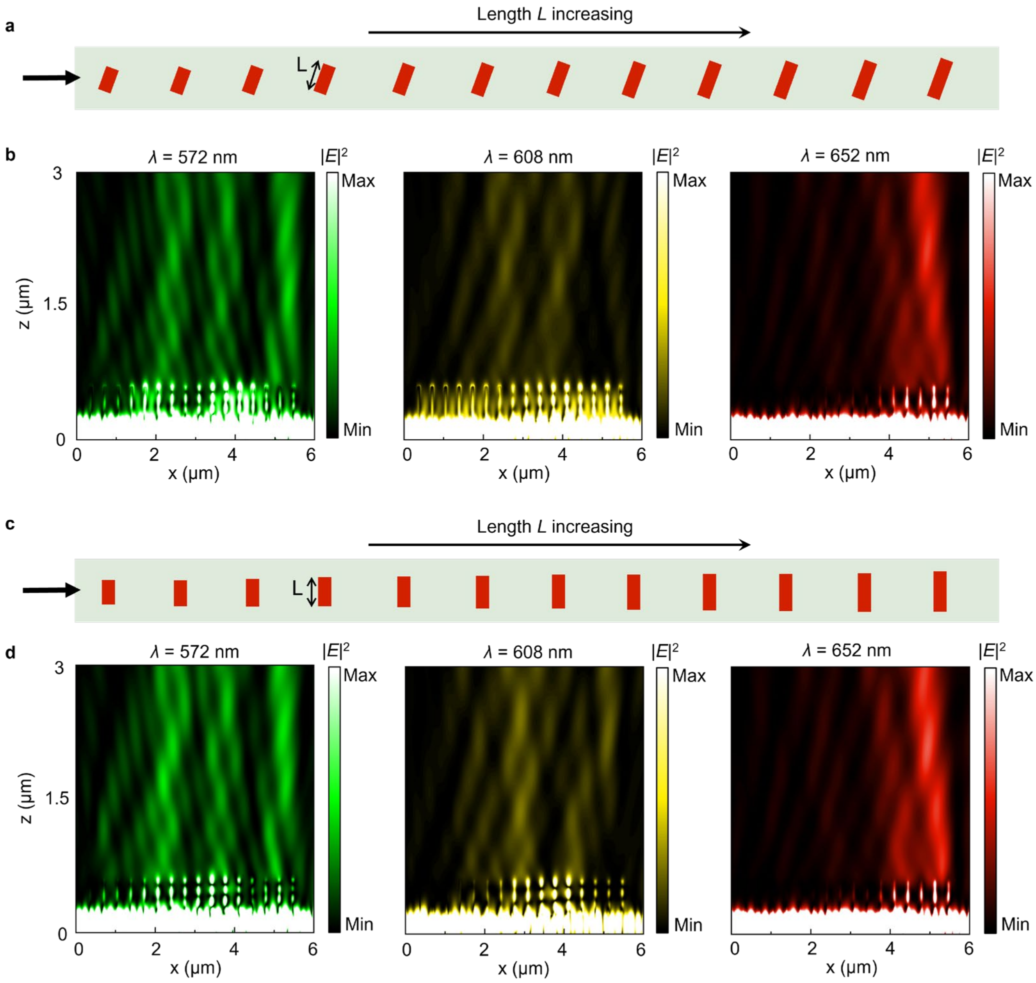

Fig. S11 Numerical simulations of conventional on-chip grating out-couplers for extracting guided waves. (a) Schematic of a conventional on-chip grating out-coupling array with the meta-atom (tilting angle $\theta = 20°$) length L increasing from 95 nm to 170 nm (keeping the width fixed at 60 nm) along the x-direction. (b) Simulated electric-field intensity ($|E|^2$) profiles extracted by the gradient array in (a) from the waveguide at the wavelengths of 572 nm, 608 nm, and 652 nm. The black arrows represent the propagation direction of the guided waves. (c) Schematic of a conventional on-chip grating out-coupling array with the meta-atom (tilting angle $\theta = 0°$) length L increasing from 95 nm to 170 nm (keeping the width fixed at 60 nm) along the x-direction. (d) Simulated electric-field intensity ($|E|^2$) profiles extracted by the gradient array in (c) from the waveguide at the wavelengths of 572 nm, 608 nm, and 652 nm.

Here, we numerically simulated the extraction performance of conventional on-chip grating out-couplers with the same continuous dimensional gradient variation as in Fig.

S10. First, we remove one nanoblock in the meta-diatomic structure and then perform numerical simulations, as shown in Fig. S11a. Compared with the q-BIC-assisted meta-diatom array, such a conventional GO does not exhibit any wavelength-selective extraction and routing performance in the broadband visible light range (λ < 645 nm) (Fig. S11b), except for the weak extraction of the large-sized nanoblocks at long wavelengths. Then, we further set the meta-atom tilting angle in Fig. S11a to 0° for numerical simulation, as shown in Fig. S11c. The simulated electric-field intensity $|E|^2$ profiles at the *x-z* cross-section in Fig. S11d are consistent with those in Fig. S11b and do not perform selective extraction.

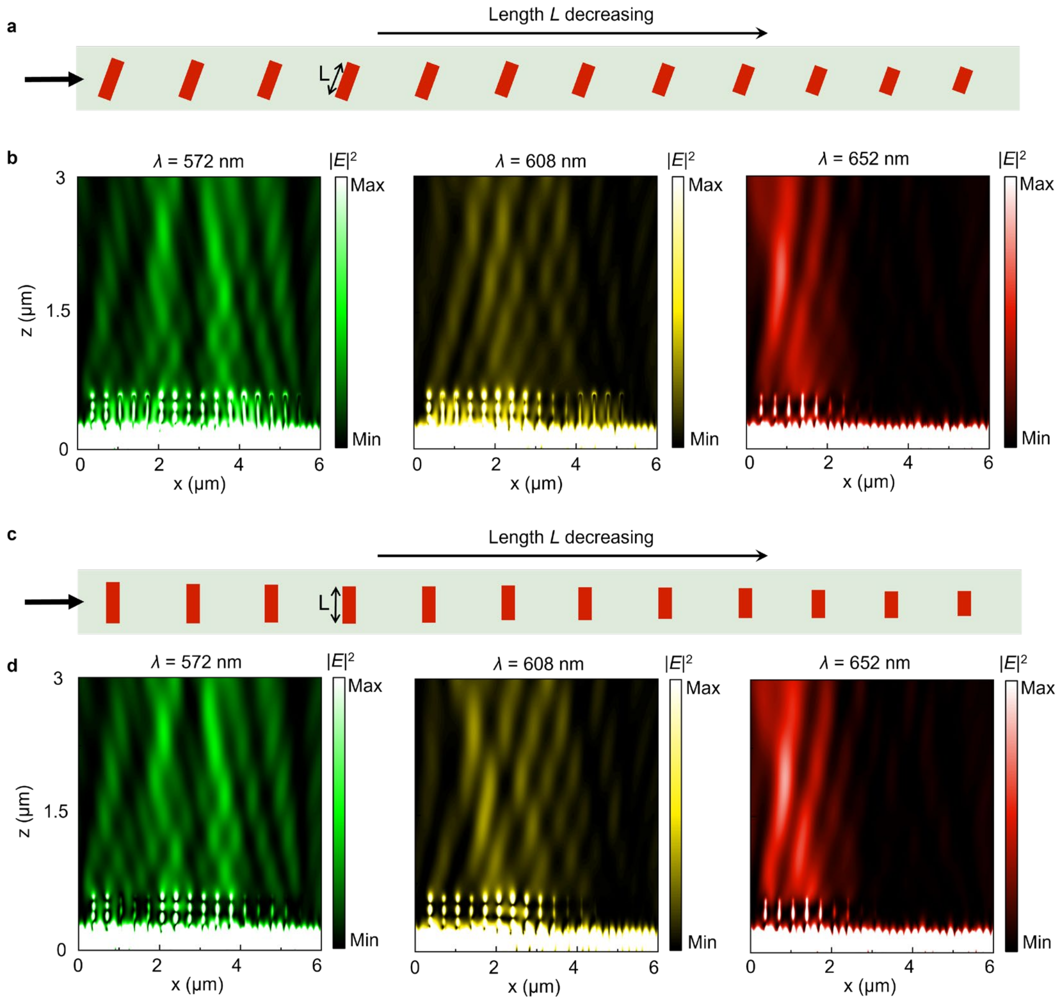

Fig. S12 Numerical simulations of conventional on-chip grating out-couplers for extracting guided waves. (a) Schematic of a conventional on-chip grating out-coupling array with the meta-atom (tilting angle $\theta = 20°$) length L decreasing from 170 nm to 95 nm (keeping the width fixed at 60 nm) along the x-direction. (b) Simulated electric-field intensity ($|E|^2$) profiles extracted by the gradient array in (a) from the waveguide at the wavelengths of 572 nm, 608 nm, and 652 nm. The black arrows represent the propagation direction of the guided waves. (c) Schematic of a conventional on-chip grating out-coupling array with the meta-atom (tilting angle $\theta = 0°$) length L decreasing from 170 nm to 95 nm (keeping the width fixed at 60 nm) along the x-direction. (d) Simulated electric-field intensity ($|E|^2$) profiles extracted by the gradient array in (c) from the waveguide at the wavelengths of 572 nm, 608 nm, and 652 nm.

In addition, we numerically simulated the extraction performance of conventional on-chip GO with an opposite continuous dimensional gradient variation (length L decreasing from 170 nm to 95 nm) compared to Fig. S11, as shown in Fig. S12a and Fig. S12c. The corresponding electric-field intensity $|E|^2$ profiles in Fig. S12b and Fig. S12d also exhibit the performance of no wavelength-selective extraction and routing in the broadband visible range.

In addition, we simulated and compared the extraction performance of the out-coupling grating with distinct structural parameters (period P = 300 nm), length L increasing from 50 nm to 220 nm (keeping the width fixed at 50 nm), as shown in Fig. S13. The simulated electric-field intensity profiles are consistent with those in Fig. S10-S12 and illustrate that the q-BIC-assisted metasurface design exhibits the capability of wavelength-selective extraction and routing compared to conventional grating-based out-couplers.

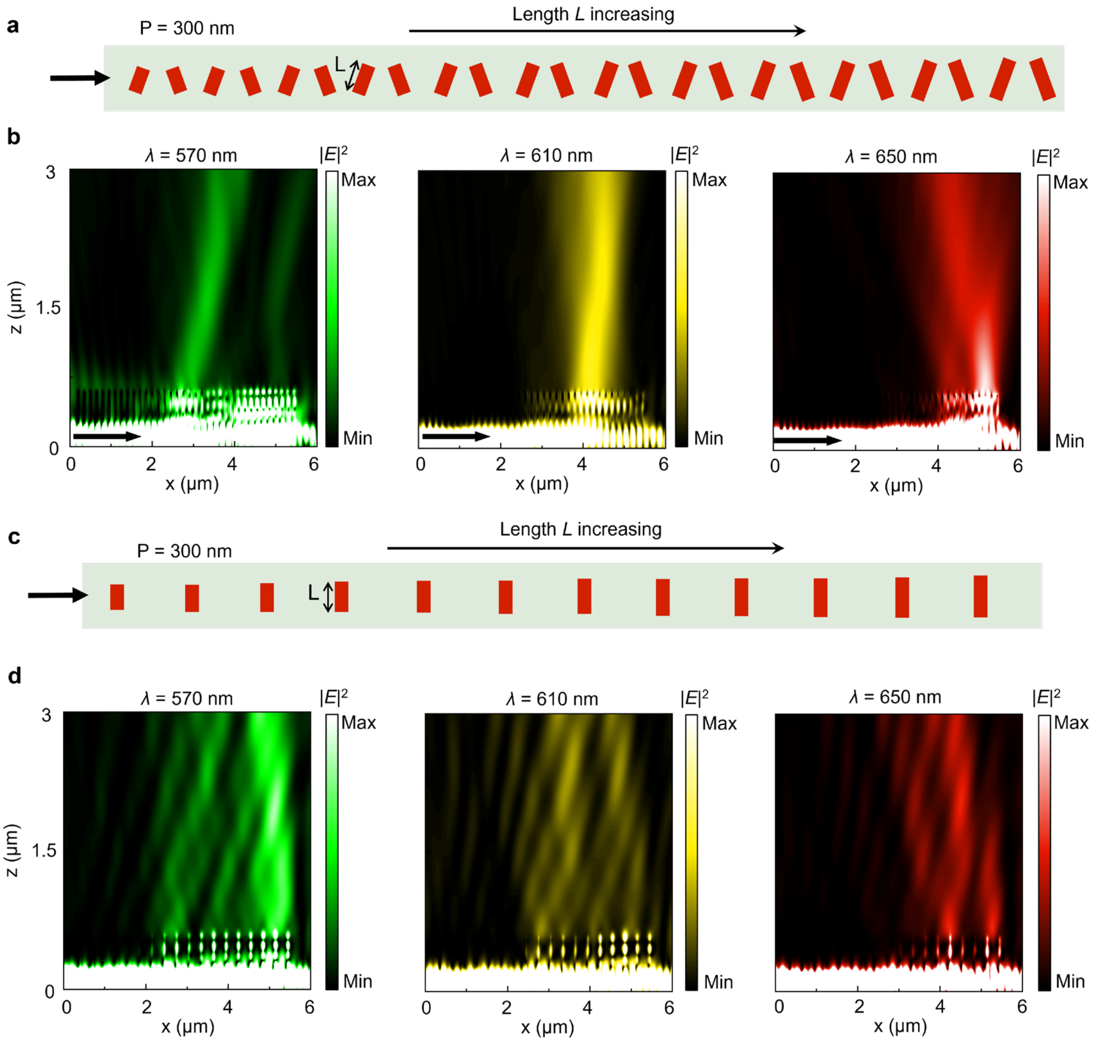

Fig. S13 Numerical simulation comparison of on-chip q-BIC-assisted metasurfaces and grating-based array for guided wave extraction. (a) Schematic of an on-chip q-BIC-assisted continuous gradient array with the meta-diatom (tilting angle $\theta = 20°$ and period $P = 300$ nm), length L increasing from 50 nm to 220 nm (keeping the width fixed at 50 nm) along the x-direction. (b) Simulated electric-field intensity ($|E|^2$) profiles extracted by the gradient array in (a) from the waveguide at the wavelengths of 570 nm, 610 nm, and 650 nm. The black arrows represent the propagation direction of the guided waves. (c) Schematic of a conventional on-chip grating out-coupling array with the meta-atom (tilting angle $\theta = 0°$) length L increasing from 50 nm to 220 nm (keeping the width fixed at 50 nm) along the x-direction. (d) Simulated electric-field intensity ($|E|^2$) profiles extracted by the gradient array in (c) from the waveguide at the wavelengths of 570 nm, 610 nm, and 650 nm.

S7. Numerical simulations of on-chip q-BIC-assisted metasurfaces with tilting angle gradient variation for guided wave extraction

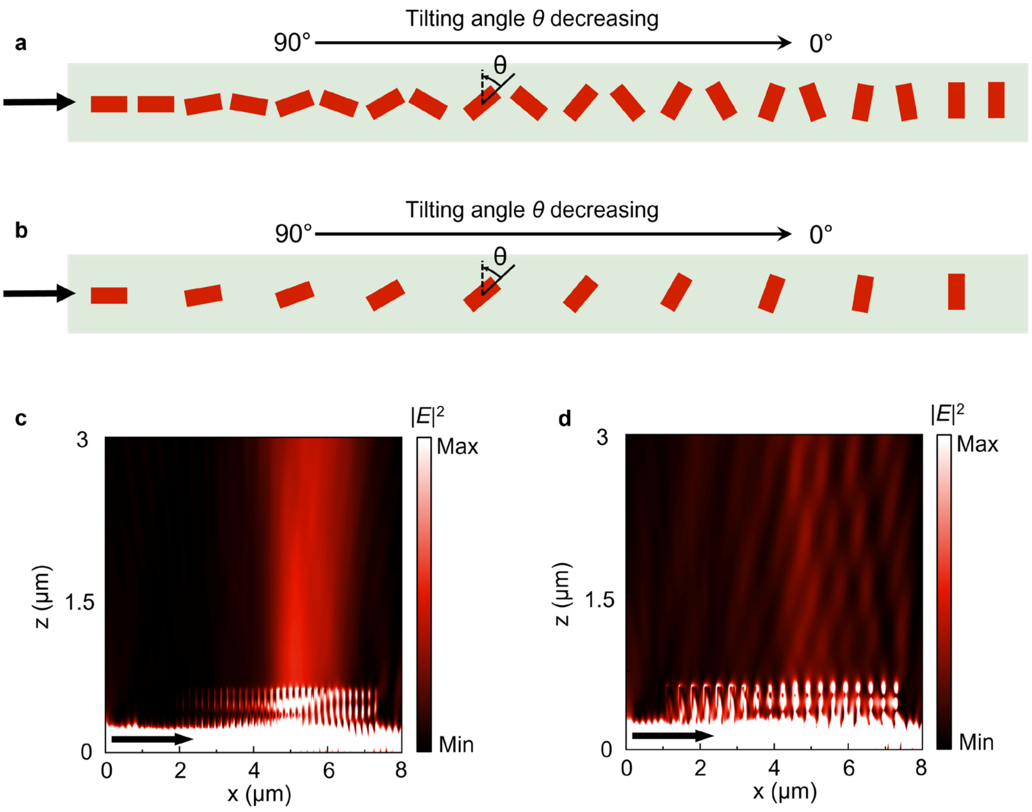

Fig. S14 (a) Schematic of an on-chip q-BIC-assisted continuous gradient array with the meta-diatom tilting angle θ decreasing from 90° to 0° along the x-direction. (b) Schematic of a conventional on-chip grating out-coupling array with the meta-atom tilting angle θ decreasing from 90° to 0° along the x-direction. (c) Simulated electric-field intensity ($|E|^2$) profiles extracted by the gradient array in (a) from the waveguide at the wavelength of 608 nm. The black arrows represent the propagation direction of the guided waves. (d) Simulated electric-field intensity ($|E|^2$) profiles extracted by the gradient array in (b) from the waveguide at the wavelength of 608 nm.

In Fig. S14a, we numerically simulated the extraction performance of on-chip q-BIC-assisted metasurfaces with tilting angle gradient variation (θ decreasing from 90° to 0°). The corresponding electric-field intensity $|E|^2$ profile is exhibited in Fig. S14c, and it is observed that when the wavelength of 608 nm (extraction peak) is incident, the out-coupling intensity reaches the maximum at the position where the meta-diatom tilting angle approaches ~ 20°. However, when a single meta-atom is removed from the meta-

diatom (Fig. S14b), the resulting simulated intensity distribution (Fig. S14d) demonstrates no significant enhancement in out-coupling at the tilting angle of ~ 20°. This also further demonstrates the ability of our q-BIC-assisted meta-diatom array to tune the intensity of the extracted guided waves.

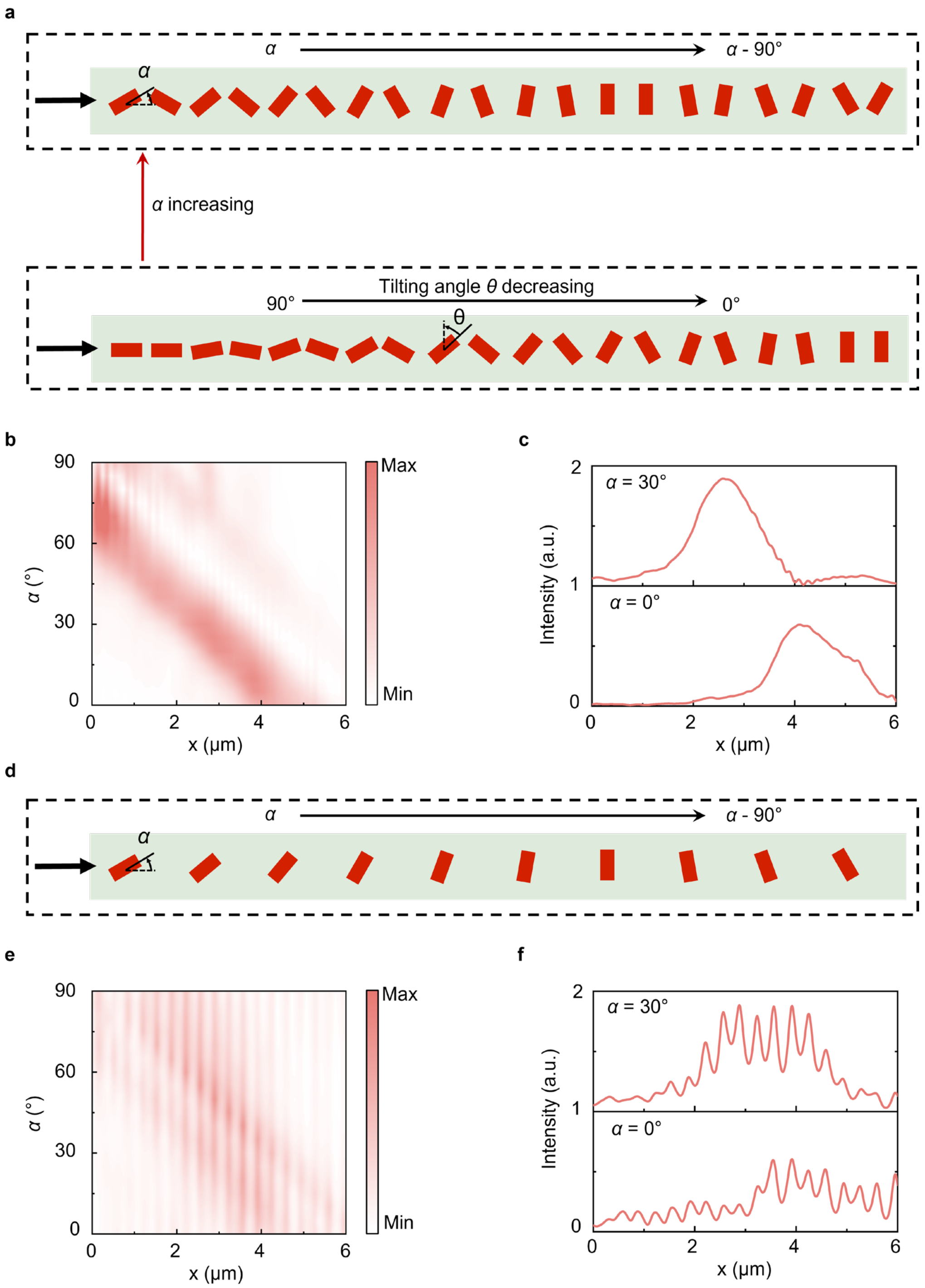

Fig. S15 (a) Schematic of an on-chip q-BIC-assisted continuous gradient array with the meta-diatom tilting angle θ decreasing from α to α - 90° along the x-direction. Here, α (varying from 0° to 90°) represents the tilting angle of the first pair of meta-diatoms in the gradient array with respect to the x-axis. The black arrows represent the propagation direction of the guided waves. (b) Simulated extraction intensity profile of the q-BIC-assisted gradient arrays along the x-direction at different tilting angles α (varying from 0° to 90°). The monitor is located 100 nm above the array. (c) Line plot of intensity profile for the case of α = 0° and α = 30° extracted from (b). (d) Schematic of a conventional on-chip grating out-coupling array with the meta-atom tilting angle θ decreasing from α to α - 90° along the x-direction. (e) Simulated extraction intensity profile of the conventional gradient grating along the x-direction at different tilting angles α (varying from 0° to 90°). (f) Line plot of intensity profile for the case of α = 0° and α = 30° extracted from (e).

We further investigated the extraction performance of various q-BIC-assisted meta-diatom gradient arrays, as shown in Fig. S15a. In this analysis, the tilting angle α of the initial meta-diatom pair of the array is varied from 0° to 90°, while maintaining a constant gradient (i.e., varying from α to α - 90°) across all arrays. The intensity distribution along the x-direction at varying angles α, as depicted in Fig. S15b and S13(c), reveals that for these arrays with the same angle gradient but different initial angles α, the location of the maximum intensity of the extracted light consistently corresponds to the diatomic tilt angle of ~ 20°. For comparison, we remove one of the meta-diatoms to simulate the corresponding intensity profile (Fig. S15d-f). It is observed that as the tilt angle approaches 20°, the gradient arrays composed of a single meta-atom exhibit limited intensity enhancement and a diffuse intensity profile in guided wave extraction.

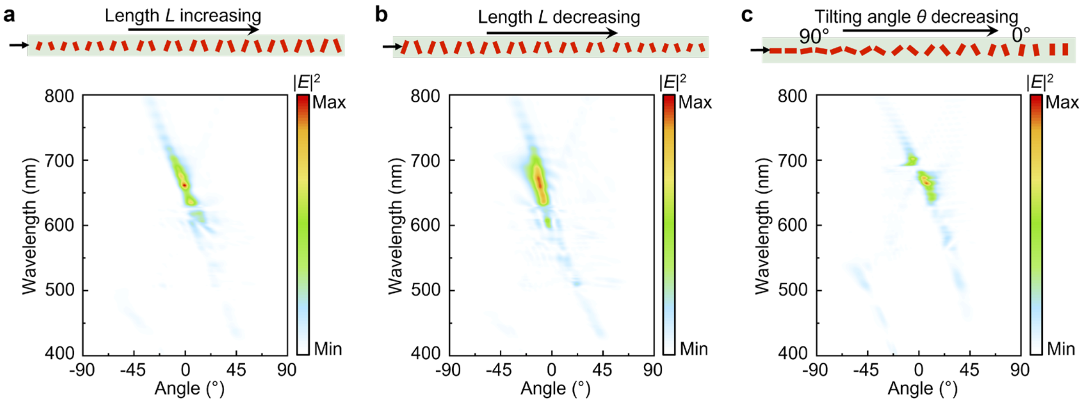

Fig. S16 Simulated far-field intensity $|E|^2$ as a function of deflection angles (x-axis) and wavelength (y-axis) for on-chip q-BIC-assisted arrays with distinct gradient structural parameter variation. (a) The on-chip continuous gradient array with the meta-diatom (tilting angle $\theta = 20°$) length L increasing. (b) The on-chip continuous gradient array with the meta-diatom (tilting angle $\theta = 20°$) length L decreasing. (c) The on-chip continuous gradient array with the meta-diatom tilting angle θ decreases from 90° to 0°.

The deflection angle observed at short-wavelength output light in Fig. 2j-l and Fig. S10-S14 is caused by the grating dispersion of the out-coupling meta-diatom array. Specifically, the length L of the gradient meta-diatom array in Fig. 2j-l increases at a relatively slow rate, allowing the array to be approximated as a periodically arranged out-coupling grating with a period of $P = 340$ nm. Therefore, guided waves of different wavelengths are extracted at distinct deflection angles that approximately satisfy the grating equation, as illustrated in Fig. S16a. The extracted red light (~ 650 nm) is emitted nearly vertically, while the green light (~ 570 nm) is extracted at a deflection angle of approximately 15°. Similarly, for the other gradient meta-diatom arrays (including gradient arrays with decreasing length and decreasing tilting angle), the deflection angle of the output light follows the same principle, as shown in Fig. S16b and S16c. Therefore, the deflection angles at short-wavelength output light in Fig. 2j-l and Fig. S10-S14 can be observed.

S8. Simulation results of wavelength-selective extraction and color routing functions at short blue wavelengths

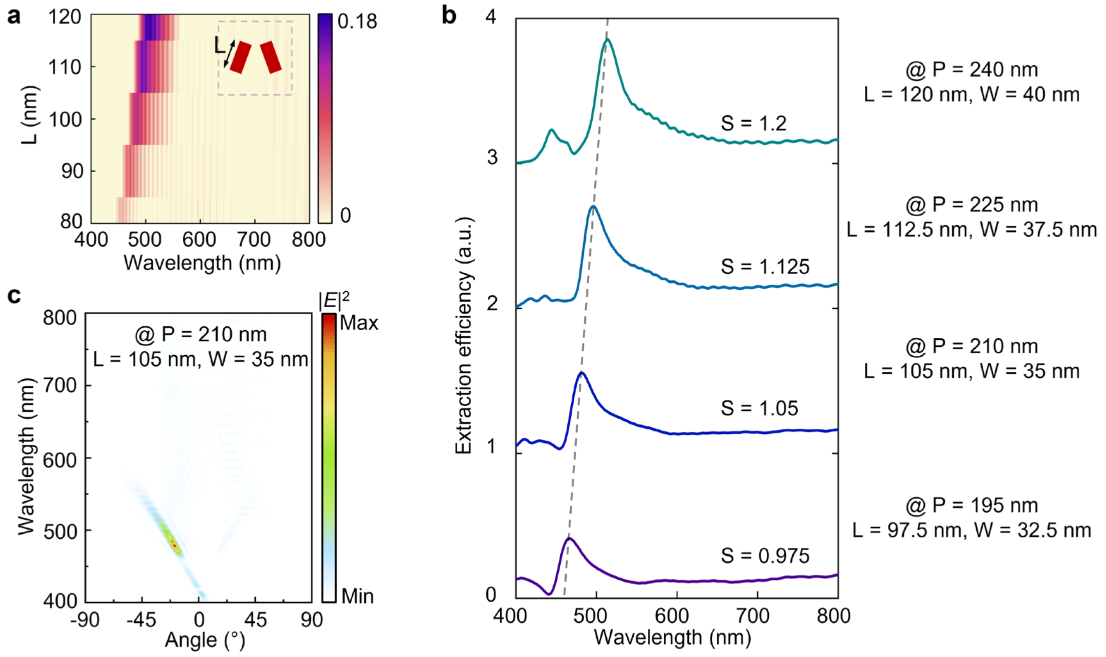

Fig. S17 Numerical simulations of on-chip q-BIC-assisted metasurfaces for blue wavelength extraction. (a) Simulated extraction spectra of the q-BIC meta-diatomic array with length L varying from 80 nm to 120 nm. The corresponding parameters are $W = 40$ nm, $P_x = P_y = 200$ nm, and H = 380 nm. (b) Line plot of extraction spectra of q-BIC meta-diatomic array with the scaling factors S (as given by $L = L_0 \times S$, $W = W_0 \times S$, $P = P_0 \times S$) varying from 0.975 to 1.2 while keeping θ fixed at 20°. The corresponding parameters are $L_0 = 100$ nm, $W_0 = 33.3$ nm, and $P_0 = 200$ nm when $S = 1$. (c) Simulated far-field intensity $|E|^2$ as a function of deflection angles (x-axis) and wavelength (y-axis). The corresponding parameters are $L = 105$ nm, $W = 35$ nm, $P_x = P_y = 210$ nm, and $H = 380$ nm.

Although we only demonstrated the extraction performance from red to green wavelengths in the main text, we can still achieve wavelength-selective extraction and color routing functions covering the blue light band in simulation, as shown in Figs. S17 and S18. Specifically, when the meta-diatomic length L increases from 80 nm to 120 nm (with a fixed width of 40 nm and period of 200 nm), the simulated extraction spectral peak position varies from 458 nm to 508 nm (Fig. S17a). We further numerically investigate the extraction spectra of different on-chip meta-diatom arrays

with the scaling factor S (as given by $L = L_0 \times S$, $W = W_0 \times S$, $P = P_0 \times S$) varying from 0.975 to 1.2 (keeping a fixed tilting angle $\theta = 20°$), as shown in Fig. S17b. As the scaling factor increases, the simulated extracted peak position redshifts from 465 nm to 512 nm, covering the blue band of the visible spectrum. Figure S17c plots the far-field intensity $|E|^2$ as a function of the deflection angle and wavelength of the out-coupling light for the on-chip q-BIC-assisted array, and it is observed that the on-chip non-local metasurface can selectively extract narrowband blue guided waves into free space. However, our current experimental demonstrations do not cover the blue part of the visible spectrum; on one hand, it is because the period and feature size of the on-chip meta-diatom structure designed for blue light extraction are relatively small, presenting significant fabrication challenges with our current manufacturing capabilities. On the other hand, due to material absorption loss, the extraction efficiency of the designed meta-diatom array in the blue band is relatively low, making experimental measurement increasingly difficult.

To further illustrate the unique color routing capability in the blue light band, we predefine a continuous gradient of meta-diatomic length L varying from 72 nm to 122 nm (keeping W fixed at 40 nm and the tilting angle θ fixed at 20°) to arrange the q-BIC-assisted meta-diatom pairs along the propagation direction (x-axis), as shown in Fig. S18a. The simulated electric-field intensity $|E|^2$ profiles at xz-cross sections of the gradient meta-diatom array (Figs. S18b-S18d), exhibit that guided waves with different blue light wavelength components would be strongly extracted from respective different locations into free space. Therefore, our on-chip non-local metasurface could

achieve modulation of the primary wavelength of the out-coupling lightwave for color routing covering the entire visible spectrum, including the blue part, which could be pursued in future work.

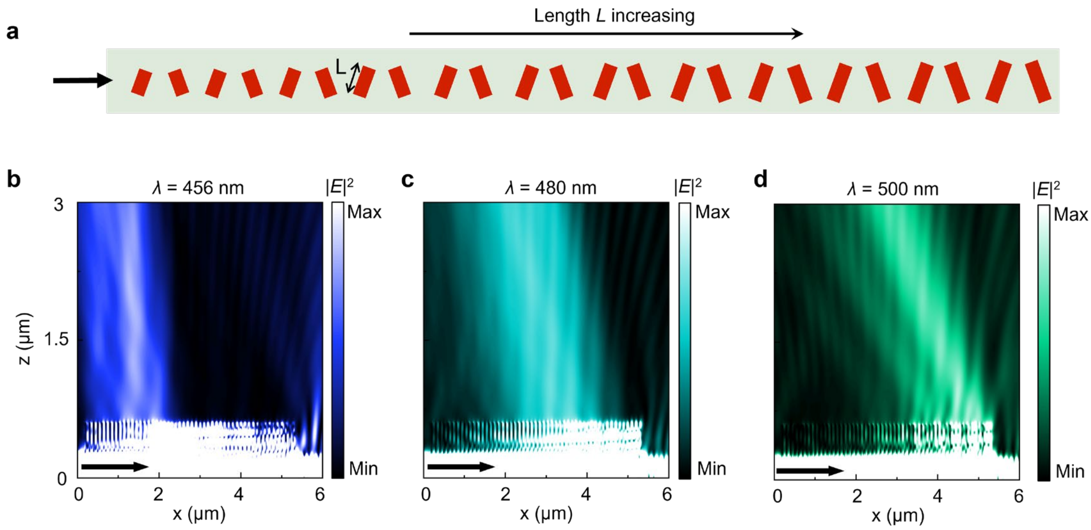

Fig. S18 Numerical simulations of on-chip q-BIC-assisted metasurfaces for blue wavelength routing. (a) Schematic of an on-chip q-BIC-assisted continuous gradient array with the meta-diatom (tilting angle θ = 20° and period P = 200 nm) length L increasing from 72 nm to 122 nm (keeping the width fixed at 40 nm) along the x-direction. (b-d) Simulated electric-field intensity ($|E|^2$) profiles extracted by the gradient array in (b) from the waveguide at the wavelengths of 456 nm, 480 nm, and 500 nm. The black arrows represent the propagation direction of the guided waves.

S9. Extraction performance of the meta-diatomic hole array structure etched on the silicon nitride waveguide

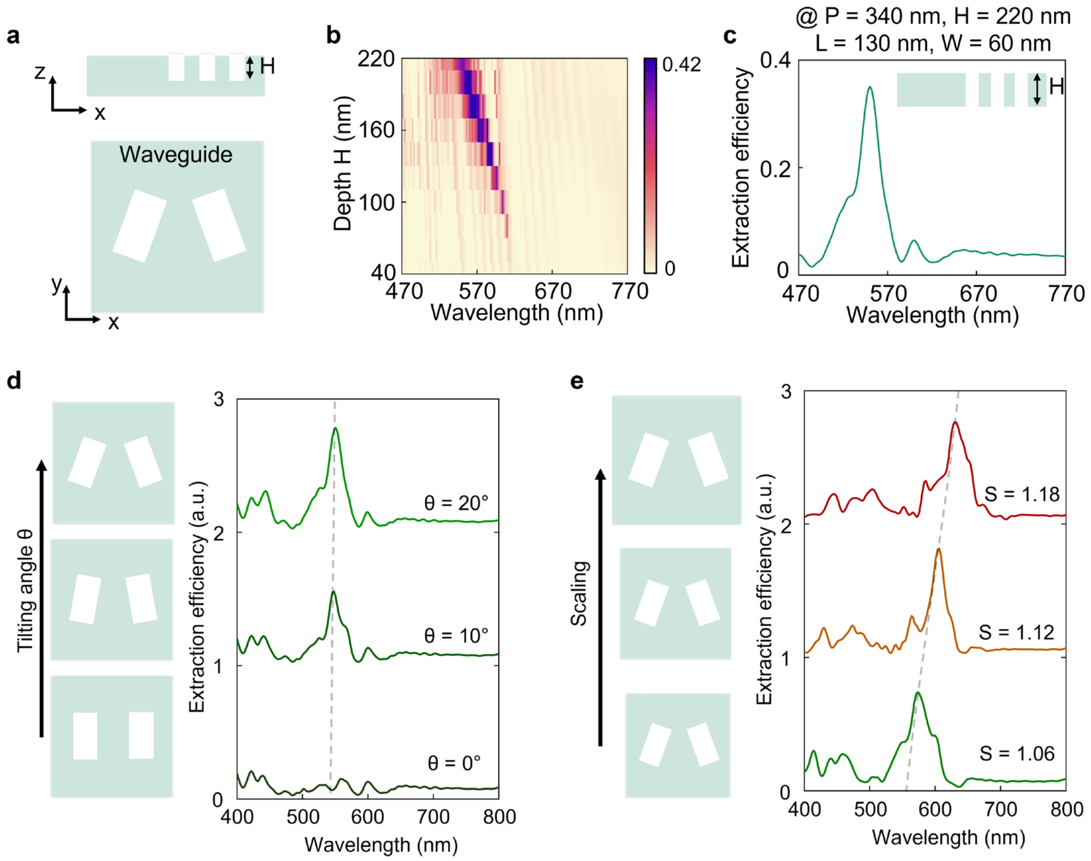

Fig. S19 Numerical analysis of the on-chip meta-diatomic rectangular hole array for wavelength extraction and intensity tuning. (a) Schematic of on-chip diatomic pixels consisting of two tilted nanoholes. The corresponding parameters are L = 130 nm, W = 60 nm, and P_x = P_y = 340 nm. The thickness of Si_3N_4 is 220 nm. (b) Simulated extraction spectra corresponding to distinct meta-diatomic hole arrays with etching depths varying from 40 nm to 220 nm. (c) Extraction spectrum of the meta-diatomic hole array with an etching depth of 220 nm. (d) The simulated spectra of extracted light from different meta-diatomic hole pairs with the tilting angle θ varying from 0° to 20°. The corresponding parameters are L = 130 nm, W = 60 nm, H = 220 nm, and P_x = P_y = 340 nm. (e) The simulated extraction spectra from different meta-diatomic hole pairs with the scaling factor S varying from 1.06 to 1.18. The corresponding parameters are L_0 = 160 nm, W_0 = 60 nm, and P_0 = 400 nm when S = 1.

Here, we further numerically simulate nonlocal metasurfaces with etched rectangular holes in silicon nitride waveguides and study the extracted spectral properties, as shown in Fig. S19a. First, we numerically studied the extraction spectra of on-chip meta-diatomic rectangular hole arrays with etching depths varying from 40 nm to 220 nm, as

shown in Fig. S19b. It can be seen that as the etching depth increases, the extraction peak wavelength gradually blueshifts. Specifically, when the etching depth is 220 nm (corresponding to the waveguide thickness), the peak wavelength is 550 nm, and the bandwidth is ~ 28 nm (Fig. S19c). Therefore, it is feasible to directly etch silicon nitride to create on-chip metasurfaces for the narrowband spectral extraction application.

Next, we simulated the extraction spectra of different meta-diatomic hole pairs with the tilting angle θ varying from 0° to 20°, as shown in Fig. S19d, exhibiting a corresponding increase in extraction intensity as the tilting angle θ increases. In addition to the intensity control, the spectral peak can also be simultaneously tuned by tailoring the meta-diatom dimension (scaling factor S), as illustrated in Fig. S19e, which reveals that adjusting S from 1.06 to 1.18 for θ = 20° shifts the spectral peak from 572 nm to 630 nm. Overall, by elaborately engineering the geometric asymmetry and scaling factor of the meta-diatomic hole pairs, we can also achieve simultaneous modulation of extraction intensity and primary wavelength of the out-coupling lightwave.

S10. Extraction performance of on-chip meta-atom arrays with different designs on the Si_3N_4 waveguide

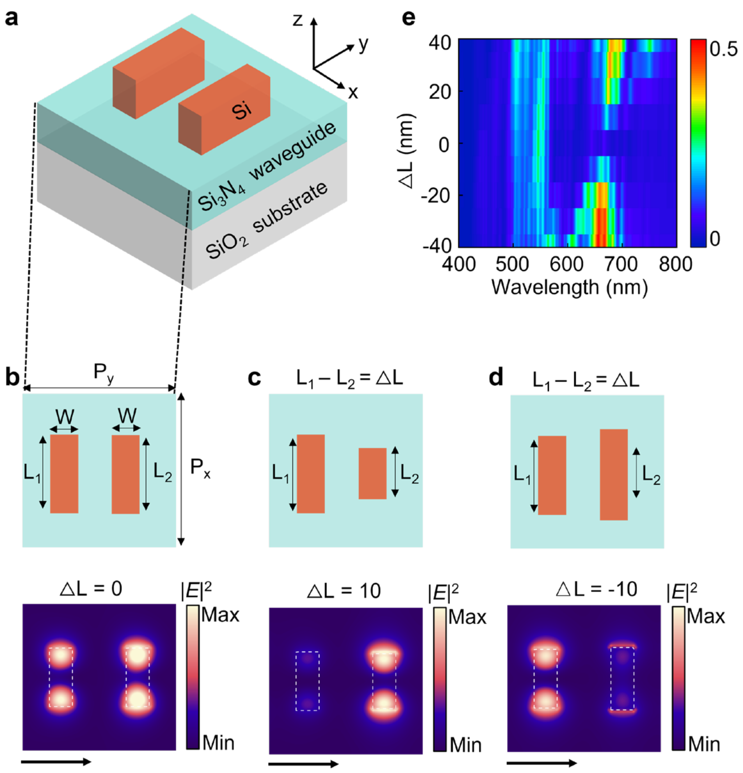

Fig. S20 (a) 3D schematic of on-chip diatomic pixels consisting of two parallel nanoblocks. (b-d) Schematic of three different length difference $\triangle L$ between two nanoblocks in the unit cell. The width W is fixed at 60 nm, and the length differences are (b) $\triangle L = 0$ nm, (c) $\triangle L = 10$ nm, and (d) $\triangle L = -10$ nm, respectively. The period is 400 nm, and the displacement between the two nanoblocks is 200 nm. Here, $\triangle L = L_1 - L_2$. Bottom: the corresponding simulated electric-field intensity ($|E|^2$) distributions in the xy-plane. The black arrows represent the propagation direction of the guided waves. (e) Simulated extraction spectra of the on-chip q-BIC meta-diatomic array as the length difference $\triangle L$ between the two nanoblocks varies from -40 nm to 40 nm.

Here, we further numerically simulated the on-chip diatom arrays consisting of two parallel nanoblocks in the unit cell, as shown in Fig. S20a. Specifically, the widths of both nanoblocks are fixed at 60 nm, and the length of the left nanoblock is fixed at 160 nm, while only the length of the right nanoblock is varied. The length difference $\triangle L$ between two nanoblocks is $L_1 - L_2$. When the length difference $\triangle L$ is 0, that is,

the lengths of the two nanoblocks are equal, and the extracted light resonates simultaneously along the long axes of both nanoblocks, as shown in the simulated electric field distribution in Fig. S20b. In contrast, when the lengths of the two nanoblocks are different, as shown in Fig. S20c and S20d, the extracted guided light exhibits strong resonance predominantly with a single nanoblock in each case. The extraction spectra of the on-chip meta-diatom arrays shown in Fig. S20e demonstrate that as the length difference $\triangle L$ varies from 0 nm to 40nm (or -40), the intensity of extracted lights increases accordingly, accompanied by a redshift (or blueshift) of the extraction peak. Therefore, this structural design is also capable of extracting guided waves into free space, exhibiting a certain degree of narrowband extraction performance and tunable intensity. However, compared with our proposed tilted diatomic q-BIC structural design, the extracted spectra (Fig. S20e) corresponding to the structure in Fig. S20a exhibit a stronger background light in the short-wavelength range (500 - 550 nm). Furthermore, due to the size-dependent intensity modulation, the modulation range is relatively limited, and the structure is more sensitive to dimensional deviations introduced during fabrication.

In addition, we also numerically simulated the extracted spectra of three different on-chip single-atom arrays, each consisting of a single nanoblock, as shown in Fig. S21a-c. However, the corresponding extraction spectra presented in Fig. S21d-f do not exhibit regular guided wave extraction performance with narrow bandwidth and tunable intensity. Nevertheless, we hold that on-chip single-atom guided mode resonance

structures with narrowband extraction and intensity tunable performance may also be achieved through finer and more extensive parameter sweeps, but these would require significant computational effort and time consumption.

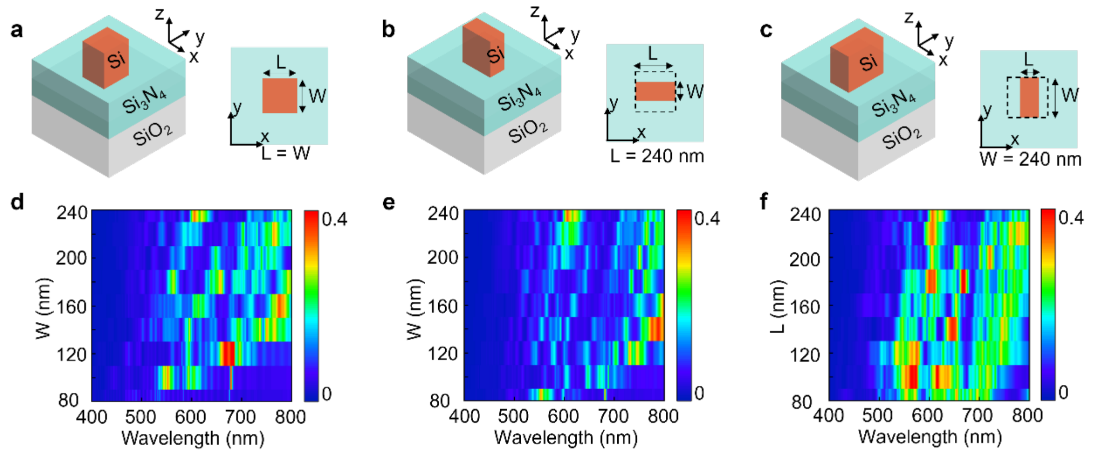

Fig. S21 (a) Schematic of an on-chip single-atom pixel consisting of a single square nanoblock. (b) Schematic of an on-chip single-atom pixel composed of a single rectangular nanoblock. The nanoblock length is fixed at 240 nm, and the width varies from 80 nm to 240 nm. (c) Schematic of an on-chip single-atom pixel composed of a single rectangular nanoblock. The nanoblock width is fixed at 240 nm, and the length varies from 80 nm to 240 nm. (d) Simulated spectra of the on-chip arrays composed of the unit cell in (a), and the nanoblock length (or width) varies from 80 nm to 240 nm. (e) Simulated spectra of the on-chip arrays composed of the unit cell in (b). (f) Simulated spectra of the on-chip arrays composed of the unit cell in (c). The guided waves propagate along the *x* direction.

We also compare our q-BIC meta-diatomic structure and guided mode resonance structure composed of low-loss polymers for extracting guided waves. Specifically, we conducted numerical simulations of an on-chip meta-atom array composed of low-loss polymer (PMMA) nanoblocks integrated onto a Si_3N_4 waveguide, as illustrated in Fig. S22a. The array features a square lattice with a fixed period of $P_x = P_y = 400$ nm, with each PMMA nanoblock designed as a square in shape (equal length and width). As the reviewer pointed out, in such GMR systems, the structural perturbation directly influences the extraction efficiency of the out-coupling mode. This is confirmed in our simulations: as shown in Fig. S22b and S22c, as the PMMA meta-atom width W (or

length *L*) increases from 50 nm to 230 nm, the extracted intensity increases correspondingly. Additionally, the extraction spectrum exhibits a sharp resonance peak centered around 680 nm. On the other hand, by fixing the width *W* at 230 nm and sweeping the period from 340 nm to 420 nm, the extracted resonance wavelength shifts from 601 nm to 705 nm, as presented in Fig. S22d. This confirms that the period of the structure controls the resonance wavelength. These results highlight the different mechanisms in GMR-based and q-BIC-based metasurfaces for controlling light extraction. Compared with our proposed tilted diatomic q-BIC structural design, although the extraction efficiency of the GMR-based structure (~0.15) is lower than that of the BIC-based design, it exhibits a narrower extraction bandwidth (~3 nm) and a higher quality factor (Q ~226). Therefore, GMR-based designs can serve as an alternative and effective approach for controlling both the amplitude and wavelength of extracted guided waves. Such a meaningful discussion and exploration could open a valuable direction and be further investigated in our future work.

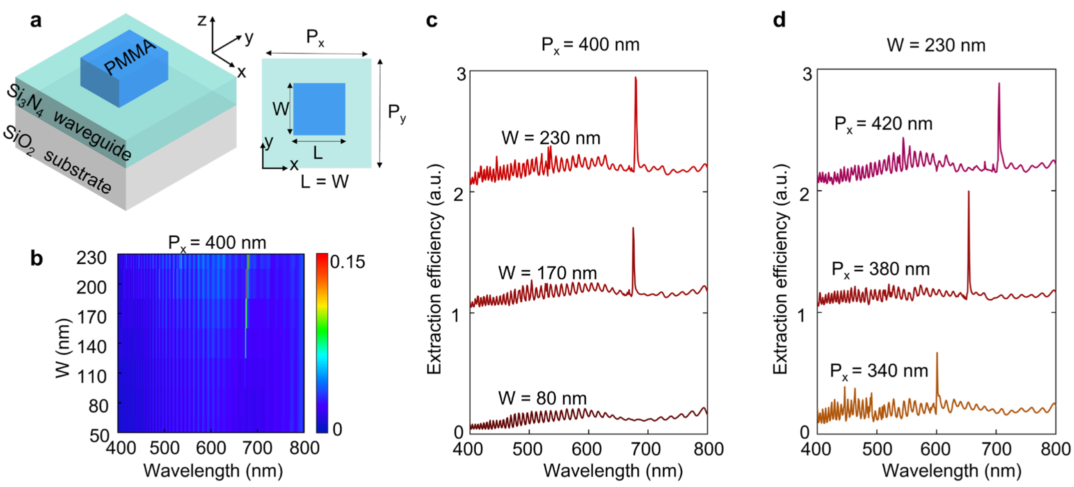

Fig. S22 Numerical analysis of the on-chip periodic array composed of low-loss polymers for guided wave extraction. (a) Schematic of an on-chip single-atom pixel consisting of a single rectangular PMMA

nanoblocks. The thickness of Si_3N_4 is 220 nm, and the height of the PMMA is 380 nm. (b) Simulated extraction spectra contour of PMMA meta-atom arrays with nanoblock length (or width) varying from 50 nm to 230 nm. The corresponding parameters are $L = W$, and $P_x = P_y = 400$ nm. (c) Line plot of the extraction spectra of the PMMA meta-atomic array for the case of nanoblock length $L = 80$nm, $L = 170$ nm, and $L = 230$ nm, respectively, while keeping period $P_x = P_y = 400$ nm. (d) Line plot of the extraction spectra of the PMMA meta-atomic array for the case of period $P_x = P_y = 420$ nm, 380 nm, and 340 nm, respectively, while keeping the length $L = W$ =230 nm. The guided waves propagate along the x direction.

That said, we respectfully would like to clarify the distinct advantages and originality of our q-BIC-based design. Our structure represents a novel and original attempt to simultaneously tailor the amplitude and narrowband wavelength of the extracted guided waves on-chip via quasi-BIC. Unlike GMR systems that rely on periodicity-defined resonances and extensive parameter sweeps, our design offers more flexibility for guided wave manipulation and advanced functionalities such as color routing and enhanced EUE, as discussed in our manuscript. Therefore, while GMR structures have significant merits in terms of resonance sharpness, we believe that our q-BIC design introduces original physical insights and opens new engineering possibilities by allowing simultaneous and tunable control over both amplitude and spectral characteristics, which is nontrivial and has not been previously reported.

In addition, we further replaced the Si material with PMMA and conducted numerical simulations of the tilted diatomic q-BIC design, as shown in Fig. S23a. The extraction spectra of different PMMA meta-diatomic pairs with tilting angles θ varying from 0° to 30° (Fig. S23b and S23c), exhibit consistently low extraction efficiency (<0.035) and no prominent extraction peak as θ increases. These results indicate that, despite PMMA being essentially lossless at optical frequencies, its low refractive index renders it

unsuitable for our q-BIC design aimed at wavelength-selective extraction and intensity modulation.

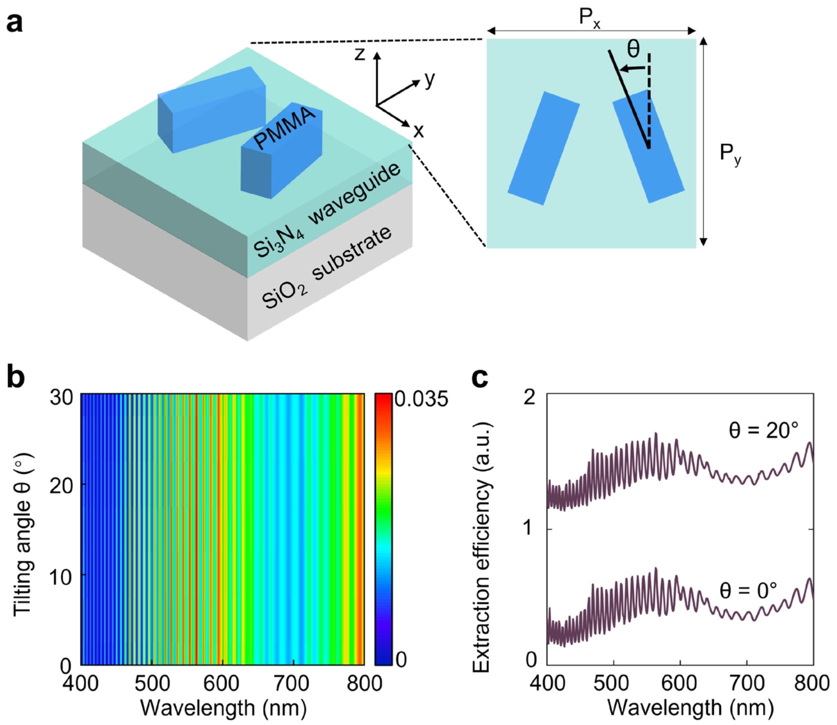

Fig. S23 Numerical analysis of the on-chip meta-diatomic array composed of low-loss polymers for wavelength extraction. (a) Schematic of on-chip diatomic pixels consisting of two tilted PMMA nanoblocks. The corresponding parameters are L = 130 nm, W = 65 nm, and $P_x = P_y$ = 400 nm. The thickness of Si_3N_4 is 220 nm and the height of the PMMA is 380 nm. (b) Simulated extraction spectra contour of PMMA meta-diatomic arrays with the tilting angle θ varying from 0° to 30°. (c) Line plot of the extraction spectra of the PMMA meta-diatomic array for the case of tilting angle θ = 0° and θ = 20°, respectively.

To further support this conclusion, we numerically compared the extraction performance of on-chip meta-diatom q-BIC arrays fabricated from different nanostructure materials, including TiO_2, GaN, Si, and PMMA (with their corresponding refractive indices shown in Fig. S24a-b). As illustrated in Fig. S24c, the Si-based meta-diatom array achieves the highest extraction efficiency, further confirming that high-refractive-index materials, such as Si, are more suitable for our nonlocal metasurface design.

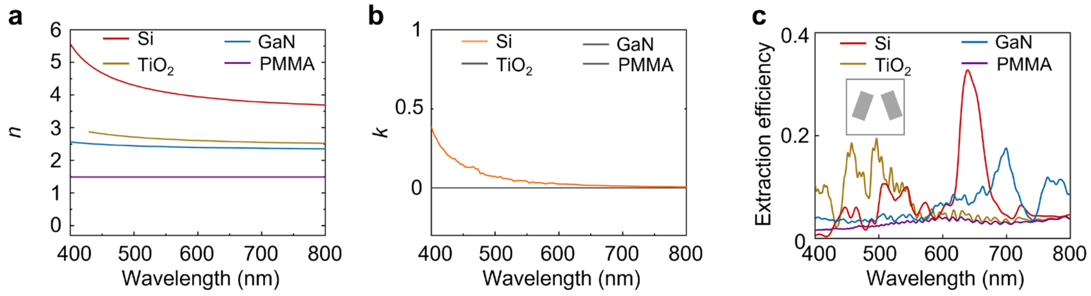

Fig. S24 (a-b) The real part n and imaginary part k of refractive indexes of TiO_2/GaN/Si_3N_4/Si/PMMA materials. (c) Simulated extraction spectra of on-chip periodic meta-diatom arrays composed of TiO_2/GaN/Si/PMMA nanostructure materials.

Overall, our proposed tilted diatomic q-BIC structural design offers the distinct advantage of enabling wavelength-selective narrowband extraction and tunable spectral intensity of the guided light through a straightforward and practical approach, without the need for extensive structural parameter scanning.

S11. The measurement methods of the extraction efficiency

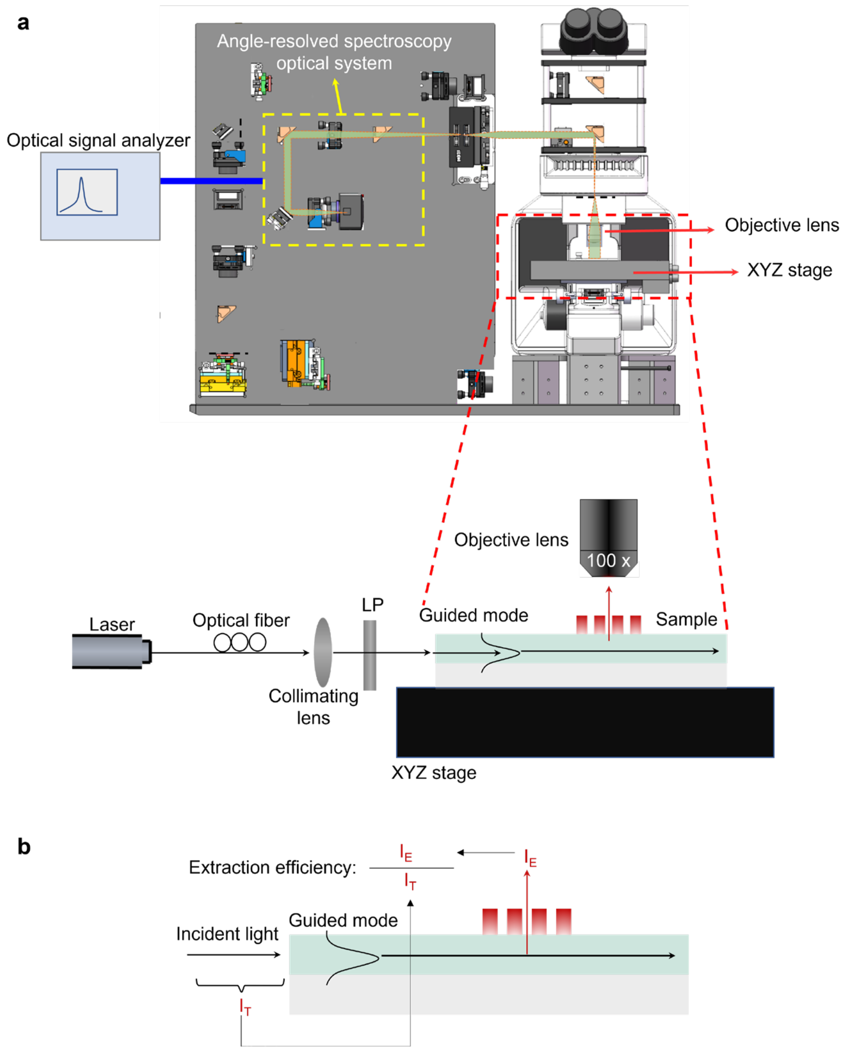

Fig. S25 (a) Schematic illustration of the optical measurement setup to measure the extraction efficiency. LP: linear polarizer. (b) Schematic of the extraction efficiency calculation of the on-chip metasurface. I_T: the total incident light intensity; I_E: the extracted light intensity.

Regarding the measurement methods of the extraction efficiency, we employed a custom-built optical setup integrating an on-chip coupling configuration and an angle-resolved spectroscopy system to evaluate the optical extraction performance of on-chip non-local metasurfaces, as illustrated in Fig. S25a. Specifically, the broadband polarized laser source (wavelength range: 500 nm - 800 nm) was first coupled into the Si_3N_4 waveguide in an end-fire manner via a fiber collimator, thereby efficiently

exciting guided modes propagating along the waveguide. Subsequently, the light extracted by the metasurface was collected utilizing a 100× objective lens with a high numerical aperture (NA = 0.9), enabling a wide angular collection range. The angularly and spatially resolved optical signals were then projected to the back focal plane of the objective and analyzed using an angle-resolved microscopic spectrometer (ARMS, Ideaoptics Inc.), which enabled Fourier-plane imaging and wavelength-resolved spectral acquisition across different extraction angles. After normalizing the incident light source intensity (I_T) in the measured spectral intensity (I_E) from the metasurface region (Fig. S25b), the final relative extraction efficiency can be obtained.

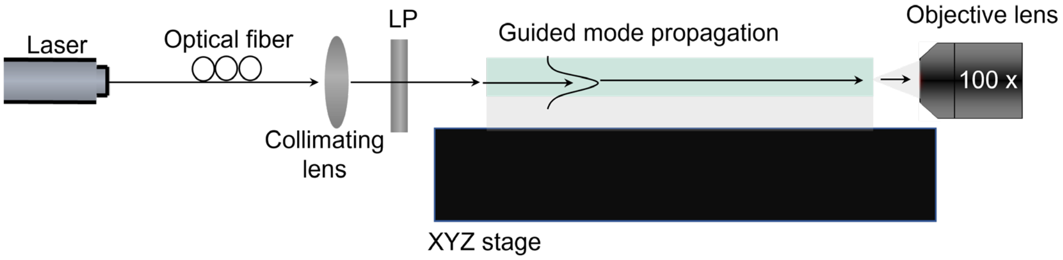

Fig. S26 Schematic illustration of the optical measurement setup to measure the coupling efficiency from the fiber into the guided mode. LP: linear polarizer.

In addition, the coupling efficiency from free space into the waveguide can be estimated based on the experimental configuration illustrated in Fig. S26. Specifically, the input optical power (P_{in}) before coupling into the waveguide is first measured utilizing an optical power meter. Subsequently, the output light is directly collected at the waveguide end facet with a high numerical aperture (NA) objective lens to measure the output optical power (P_{out}). The free-space-to-waveguide coupling efficiency is then determined by calculating the ratio (P_{out}/P_{in}) of the output optical power to the incident optical power, assuming negligible propagation losses in the optical path. Here, the

average coupling efficiency from the simulations is about 15%-20%, and the experimental coupling efficiency is estimated as about 10%-15%. Despite that the overall efficiency is not high, it is enough for on-chip narrowband spectral extraction and color routing functionality, and practically observing the multicolor meta-display images. In addition, the low coupling efficiency here does not imply low energy utilization efficiency, because energy utilization efficiency differs conceptually from both coupling and extraction efficiency. Under the assumption of minimal propagation losses, the energy utilization efficiency can reach a higher value (close to unity), thereby indicating highly efficient in-waveguide energy management and utilization.

Regarding the potential strategy to improve the optical extraction efficiency, we can utilize low-loss and high-index dielectric materials (such as Titanium dioxide, TiO_2), appropriately increase the metasurface area, or apply more precise fabrication processing. For example, in Figs. 3f and 3g of our manuscript, we numerically studied the optical extraction efficiency for on-chip periodic arrays with different numbers of meta-diatom pairs. It is observed that as the number of meta-atoms increases from 0 to 50 along the x-direction, the simulated extraction efficiency correspondingly increases, and the maximum extraction efficiency limit can be approximated to 0.5.

Regarding the potential methods to improve the end-fire coupling efficiency, on one hand, we can utilize a tapered input waveguide structure to better match the mode field between the fiber and the waveguide, thereby enhancing coupling efficiency. This

tapered section also serves as a mode converter, enabling expansion of the mode field profile. On the other hand, optical components such as fiber lenses (e.g., spherical microlenses or graded-index lenses) or free-space collimating lenses, as well as a polarization controller, can be employed to tailor the beam size and polarization state of input light, ensuring optimal alignment with the fundamental guided mode. Additionally, a high-precision translation platform can be combined to achieve accurate alignment between the optical axis of the input beam and the waveguide facet, further improving coupling efficiency.

S12. The incident white light source in the experiment

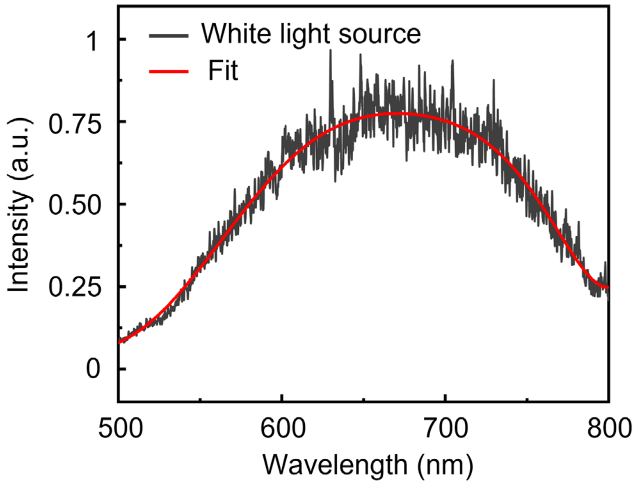

Fig. S27 The incident broadband (500 nm - 800 nm) white light source in the experiment.

In our experiment, the broadband (500 nm - 800 nm) (Fig. S27) polarized laser is coupled into the waveguide utilizing an end-fire manner with a fiber collimator.

S13. Comparison of extracted spectra under TE_0 and TE_1 mode excitation

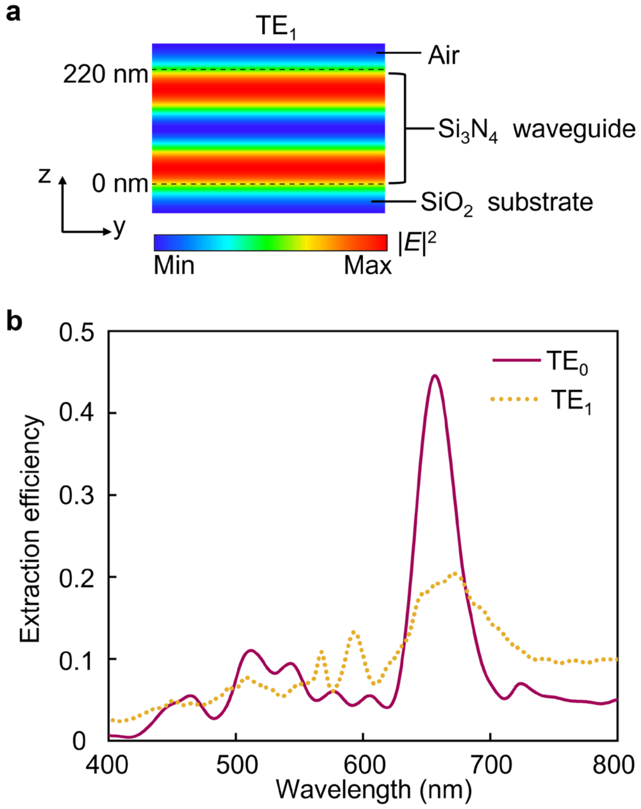

Fig. S28 (a) Guided wave incidence utilizing TE_1 mode in the simulation. The TE_1 mode guided wave propagates along the *x* direction. (b) Line plot comparison of the simulated extraction spectra when the TE_0 and TE_1 modes are incident. The corresponding parameters are L = 160 nm, W = 60 nm, $H_{waveguide}$ = 220 nm, and $P_x = P_y$ = 400 nm.

Here, according to the normalized cutoff frequency formula to calculate the cutoff conditions for each mode, we found that the primary higher-order mode supported by our on-chip waveguide configuration is the TE_1 mode, and the corresponding field distribution is shown in Fig. S28a. Compared to the TE_1 mode excitation, the extracted spectrum under the fundamental TE_0 mode exhibits a higher Q factor and stronger intensity, showing better agreement with the experimental results (Fig. S28b). Based on this observation, it can be inferred that the guided mode excited in the experiment is more likely to be the fundamental mode.

In practical measurements, to ensure that the fundamental mode of the waveguide is excited, rather than higher-order modes, during end-fired coupling from the fiber to the waveguide, a combination of the following methods can be employed. On one hand, a single-mode fiber is used as the input source, and a lens is applied to focus the fiber output beam, thereby reducing the spot size. Polarization-controlling components can be inserted into the coupling path to adjust the input polarization state, ensuring proper alignment with that of the waveguide's fundamental mode. On the other hand, a precision translation stage is utilized to finely adjust the relative position of the fiber and the sample with nanometer accuracy. This allows for accurate alignment of the fiber and waveguide axes in both lateral and vertical directions, minimizing lateral or angular offsets that could otherwise lead to the excitation of higher-order modes. Furthermore, to further minimize mode mismatch loss, a tapered waveguide structure can be introduced in the subsequent design. By gradually increasing the effective mode field size at the waveguide facet, this taper enables a smooth transition from the fiber mode to the TE_0 fundamental mode of the waveguide.

S14. Comparison of the simulated and experimental extraction spectra

Regarding the discrepancy between the experimental Q factor in Fig. 3e with the simulated Q factor in Fig. 2i, we hold that it might be caused by potential simulation inaccuracies arising from the mesh resolution limitations, material refractive index mismatch problem, and imperfect fabrication process. For instance, considering available computing resources, the limited number of meta-diatoms (N < 50) was employed along the propagation direction to balance computational efficiency in the simulations, whereas in the experiments, larger arrays (N > 100) were utilized, which may improve the Q-factor. On the other hand, in the measurements, the systematic error of the measuring device, including the post-processing error, may be another important reason for the deviation. Nevertheless, these problems may be improved and solved by applying more precise fabrication procedures, accurately calibrating the devices, or utilizing multiple measurement methods.

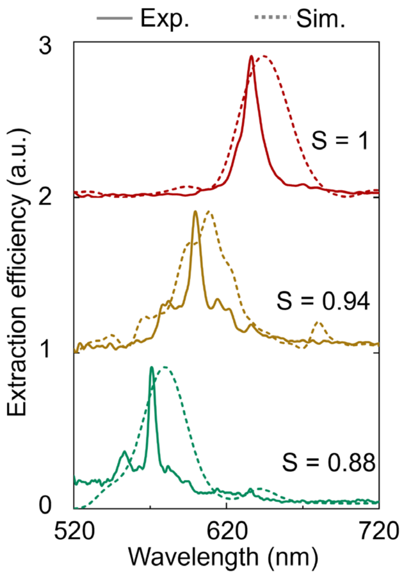

Fig. S29 The experimental and simulated extraction spectra from different meta-diatom pairs in Fig. 3e, with the scaling factor *S* varying from 0.88 to 1.

A direct comparison between the simulation and measurement results shown in Fig. 3e is provided in Fig. S29. Due to fabrication imperfections and discrepancies in the actual material refractive indices, the measured optical spectra of the fabricated structures exhibit noticeable deviations from the simulated results based on the ideal design parameters. Nevertheless, the experimental spectra show good agreement with the overall trend observed in the simulations.

S15. Numerical comparison of the extraction spectra for on-chip meta-diatoms with rectangular and rounded corners

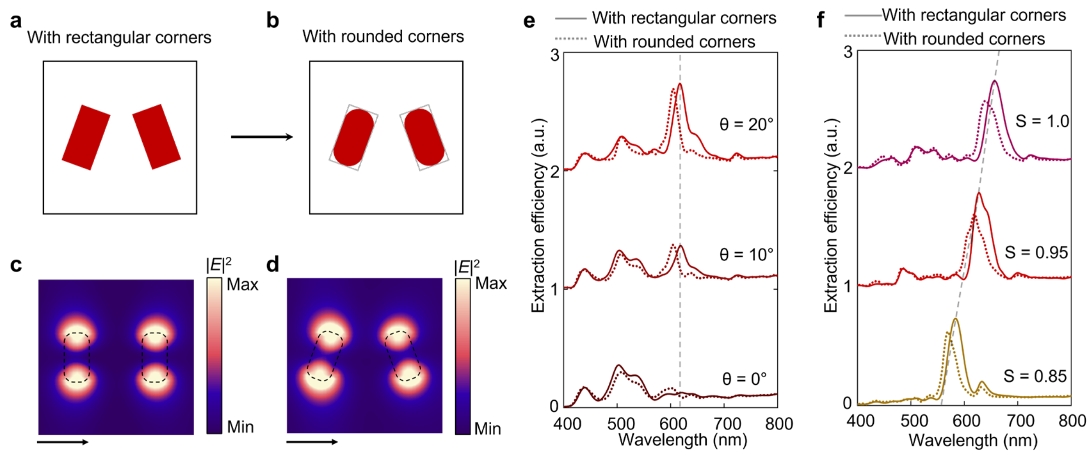

Fig. S30 Numerical comparison of extracted spectra of on-chip meta-diatom structures with rectangular and rounded corners. (a-b) Schematic of on-chip meta-diatom structures with rectangular and rounded corners. (c-d) When the TE_0 mode guided wave is incident from the x direction, the simulated electric field intensity ($|E|^2$) distribution in the xy plane for the rounded meta-diatomic structure with tilting angles $\theta = 0°$ and $\theta = 20°$. (e) Line plot comparison of extraction spectra for meta-diatom arrays with rectangular and rounded corners for the case of tilting angle $\theta = 0°$, $\theta = 10°$, and $\theta = 20°$. The Q-factors vary from 23 to 18 as the angle θ increases from 10° to 20° for rectangular-corner arrays, and the Q-factors for rounded-corner arrays vary from 34 to 30 as the angle θ increases from 10° to 20°. (f) Line plot comparison of extraction spectra for meta-diatom arrays with rectangular and rounded corners, keeping θ fixed at 20° and varying the scaling factor S from 0.85 to 1. The average Q-factors for rectangular and rounded-corner arrays are ~17 and 19.1, respectively.

We further numerically studied and compared the extracted spectra for the on-chip meta-diatoms with rectangular (Fig. S30a) and rounded (Fig. S30b) corners through FDTD simulations. The simulated in-plane electric field intensity $|E|^2$ profiles of meta-diatoms with rounded corners corresponding to $\theta = 0°$ (Fig. S30c) and $\theta = 20°$ (Fig. S30d) exhibit the mode patterns of BIC and q-BIC being excited on the periodic array, respectively. To evaluate the effect of structure ellipticity on light extraction performance in more detail, we compare the extracted spectra of meta-diatom arrays with rectangular and rounded corners within the same figure, as shown in Figs. S30e

and S25f. It can be seen that compared to the rectangular-corner diatomic array, the rounded meta-diatom array counterpart retains the intensity modulation and wavelength extraction characteristics, except for a blue shift of the extraction peak and a reduction in intensity. In addition, it is worth noting that the Q-factors of the rounded-corner meta-diatom array are increased.

Therefore, our structural design is sensitive to size variation, however, such a light geometry discrepancy between rounded and rectangular corner profiles would not strongly impact the intensity modulation and wavelength extraction functionality. This could be further improved by applying size compensation or more precise fabrication procedures.

S16. Numerical comparison of extracted spectra of on-chip meta-diatom structures with and without offset between adjacent structures, and the origin and characteristics of the secondary peak

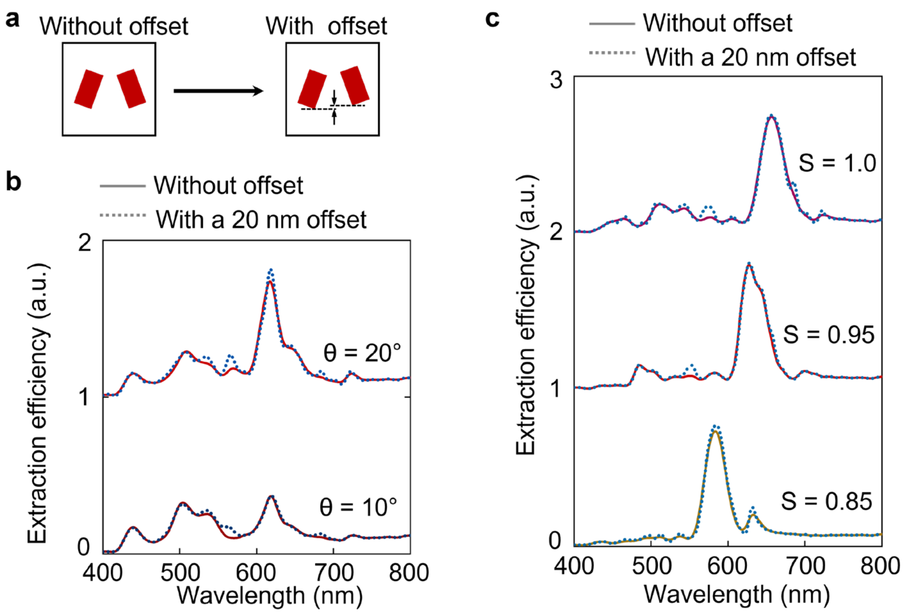

Fig. S31 Numerical comparison of extracted spectra of on-chip meta-diatom structures with and without offset between adjacent structures. (a) Schematic diagram of on-chip meta-diatom structures with and without offset. (b) Line plot comparison of extraction spectra for meta-diatom array with and without offset between adjacent structures for the case of tilting angle θ = 10° and θ = 20°. (c) Line plot comparison of extraction spectra for meta-diatom arrays with and without offset, keeping θ fixed at 20° and varying the scaling factor S from 0.85 to 1.

As observed in the SEM images (Fig. 3b), the adjacent structures exhibit a slight offset, which results in unintended asymmetry arising from fabrication imperfections. This issue can be addressed in future work by optimizing the experimental procedures. To reflect this fabrication-induced asymmetry, a structural displacement of 20 nm was incorporated into the simulation model for performance evaluation, as shown in Fig. S31a-c. Specifically, we compare the extracted spectra of meta-diatom arrays without any offset and with a 20 nm offset within the same figure, as shown in Fig. S31b and

Fig. S31c. It can be observed that, compared to the ideal meta-diatom structures without any offset, such a slight offset of 20 nm does not significantly affect the extracted peak features and the overall background level in the designed operating range. Although a weak secondary resonance appears at short wavelengths, its spectral intensity is markedly lower than that of the primary extracted peak mode, thereby exerting minimal influence on the spectral performance.

In addition, as discussed above, this weak secondary peak observed in Fig. 3c may partially result from slight structural offsets between adjacent meta-diatom elements introduced during fabrication. Nevertheless, when the tilting angle exceeds 10°, the secondary peak remains significantly weaker than the primary mode and has a negligible impact on the overall extraction performance.

On the other hand, as it is worth noting from the simulated extraction spectra in Fig. 2h and 2i, even without any structural offset, the additional peak emerges at shorter wavelengths, and this effect becomes particularly pronounced at smaller tilting angles (e.g., $\theta = 0°$) (Figure 2(h)). Furthermore, at smaller structural periods ($S = 0.85$, $P = 340$ nm in Fig. 2i), the secondary peaks become negligible or even vanish. To explain this, as shown in the far-field patterns in Fig. S7 in Section S5, we further plot the far-field intensity $|E|^2$ as a function of the deflection angle and wavelength of the out-coupling light for several on-chip q-BIC-assisted arrays with different periods and scaling factors. It is observed that while the primary modes are selectively extracted into free space, it

is accompanied by the extraction of high-order modes (m = 2), which leads to the appearance of a secondary peak at shorter wavelengths and is more pronounced at larger periods.

S17. The analysis of guided wave propagation and energy flow of the on-chip q-BIC design meta-diatom array

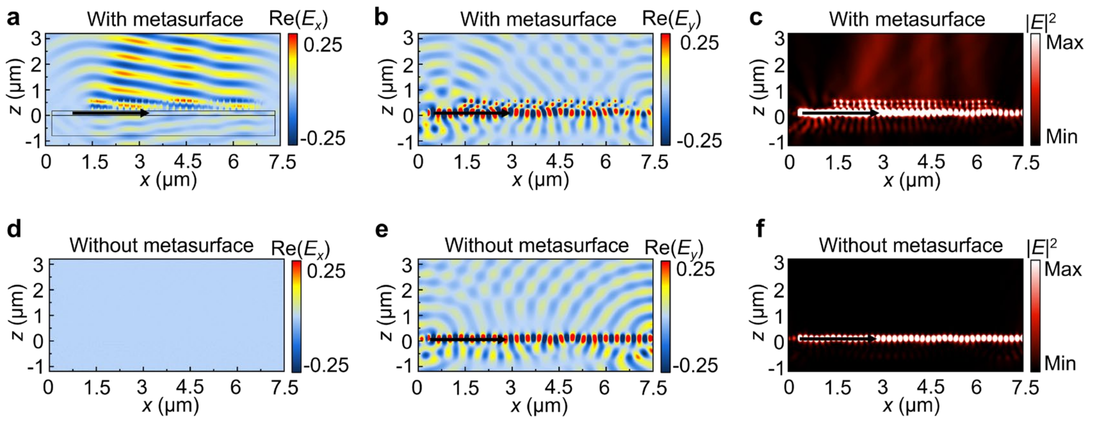

Fig. S32 Simulated guided wave propagation and extraction of the on-chip q-BIC design meta-diatom array. (a-c) Calculated corresponding electric-field E_x, electric-field E_y, and total field intensity $|E|^2$ profiles, with metasurfaces integrated onto the waveguide. (d-f) Electric-field E_x, E_y, and intensity $|E|^2$ profiles, for the waveguide without metasurface integration. The TE_0 mode guided wave is incident from the x direction. The propagation direction of the guided waves is denoted by the black arrows. The corresponding parameters are L = 130 nm, W = 65 nm, $P_x = P_y$ = 400 nm, and H = 380 nm.

To analyze and identify the origins of loss, we further numerically investigated the propagation and extraction performance of on-chip guided waves for meta-diatom arrays consisting of 14 × N-unit cells, as shown in Fig. S32a-c (the same as Fig. S4a-c in Section S2). Here, N—the row sequence number (y-direction) of the array approaches infinity due to the periodic boundary conditions in the y-direction, and the column sequence number (x-direction) is 14. It can be seen that in Fig. S32a, the propagation guided waves are primarily extracted into free space from the upper side of the metasurface, while a portion of the light is still coupled into substrate modes or leaks through the opposite (bottom) side of the substrate. Meanwhile, a significant portion of the optical energy continues to propagate along the waveguide (Figs. S32b

and S32c). Moreover, the pronounced field enhancement within the structural region indicates that part of the optical energy is absorbed by the Si material. Additionally, a small fraction of the energy is reflected backward, potentially forming standing waves or being lost due to impedance mismatch. In contrast, when no metasurface structure is integrated above the waveguide (Fig. S32d-f) (the same as Fig. S4d-f in Section S2), the guided waves remain well confined within the waveguide plane, with only minor energy leakage observed at the simulation boundaries.

S18. Comparison of the simulated extraction spectra between the on-chip q-BIC-assisted grating router and the conventional grating out-coupler

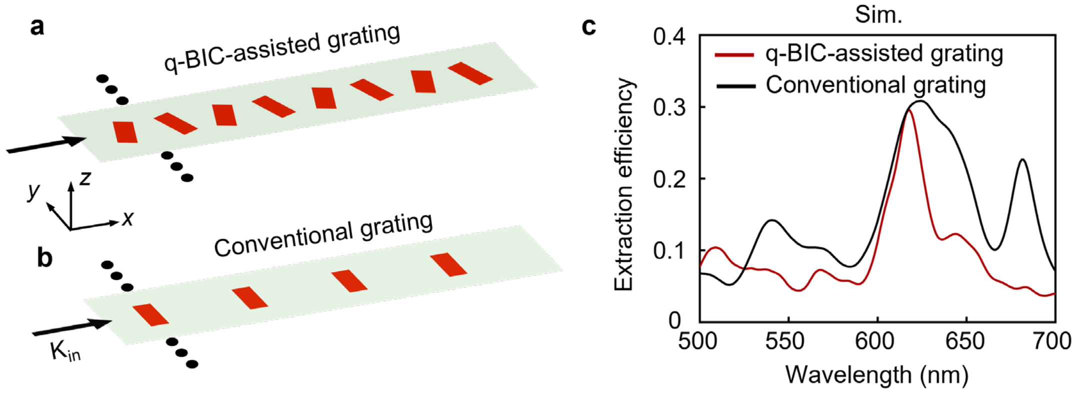

Fig. S33 (a) Schematic of an on-chip q-BIC-assisted grating array. (b) Schematic of a conventional on-chip grating out-coupling array. (c) Simulated extraction spectra profile comparison between the conventional grating out-coupler and the q-BIC-assisted router.

In simulations, we compared the extracted spectra of the conventional on-chip grating out-coupler (GO) and the q-BIC-assisted router, as shown in Fig. S33a-c. It is observed that the q-BIC-assisted grating router performs a narrowband (~ 25 nm) extraction compared to the conventional GO with a broadband (~ 56 nm) extraction characteristic.

In addition, we have performed extensive simulations involving such optimized grating structures, as shown in Fig. S34. These results indicate that while red and green light extraction (Fig. S35) can be achieved individually through careful grating design and dimensional sweeps, the extraction bandwidth remains relatively broad (typically >35 nm), the spectral response exhibits more background noise, and the intensity tuning capability is rather limited. Furthermore, such grating designs are typically highly sensitive to fabrication tolerances and involve more complex optimization processes.

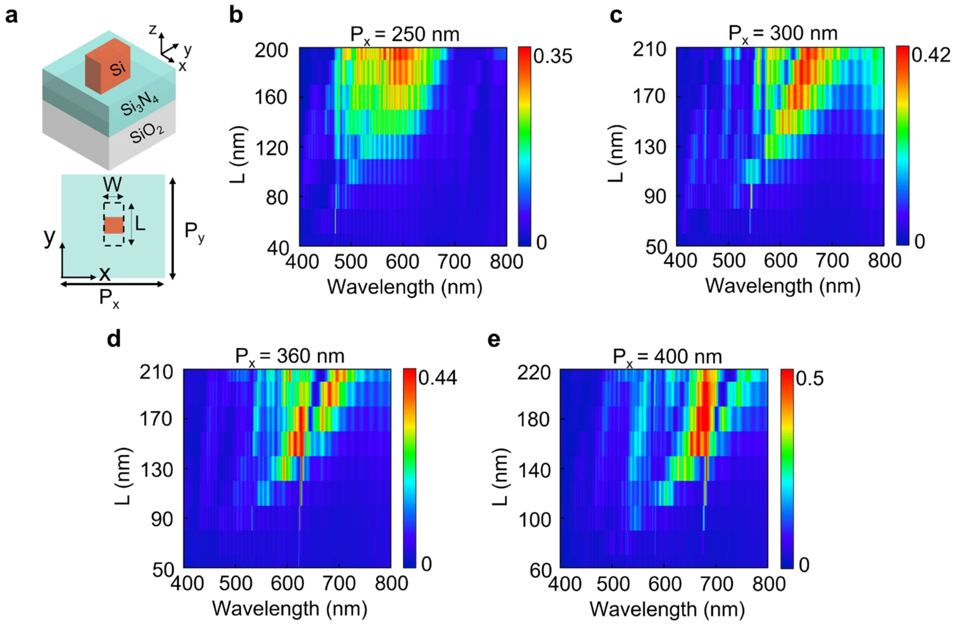

Fig. S34 (a) Schematic of an on-chip single-atom grating composed of a single rectangular nanoblock. The nanoblocks feature a fixed width and a variable length. (b) Simulated spectra of the on-chip arrays composed of the unit cell in (a). The period and nanoblock width are fixed at $P_x = P_y = 250$ nm and $W =$ 40 nm, respectively, and the length L varies from 40 nm to 200 nm. (c) Simulated spectra of the on-chip grating when the period and nanoblock width are fixed at 300 nm and 50 nm, respectively, and the length varies from 50 nm to 210 nm. (d) Simulated spectra of the on-chip grating when the period and nanoblock width are fixed at 360 nm and 50 nm, respectively, and the length varies from 50 nm to 210 nm. (e) Simulated spectra of the on-chip grating when the period and nanoblock width are fixed at 400 nm and 60 nm, respectively, and the length varies from 60 nm to 220 nm. The TE_0 guided waves propagate along the x direction.

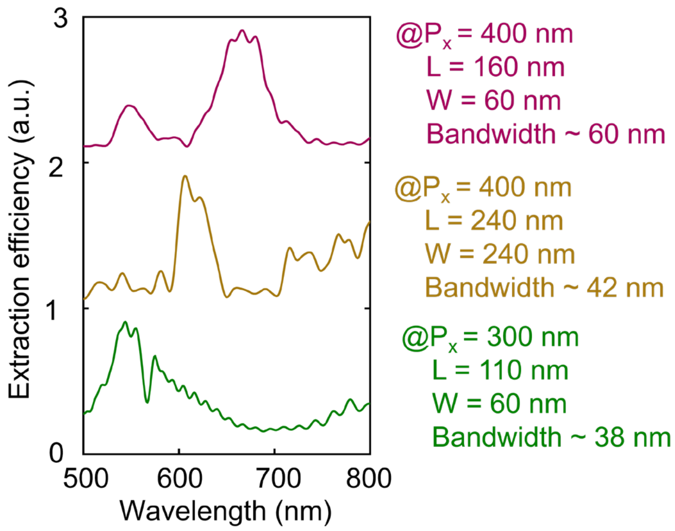

Fig. S35 The simulated extraction spectra from different single meta-atom gratings. The left side shows the corresponding structural parameters and bandwidth.

By applying the optimized grating parameters shown in Fig. S35 to design separate gratings for the red "R" and green "G" characters, they would achieve color-separated out-couplers similar to those enabled by the q-BIC design. However, we would like to emphasize that the purpose of using the grating out-coupler in our comparison (as shown in Fig. 4 in the manuscript) was not to optimize for individual extraction of red "R" and green "G" characters, but rather to serve two specific purposes. First, we intentionally used a grating with low extraction contrast between the red and green wavelengths as a reference to demonstrate the spectral overlap, which visually results in the perception of a yellow color when both red and green light are extracted simultaneously (although adopting a design with high extraction efficiency across the broad red and green wavelength bands would certainly be a better choice). This effect helps illustrate the wavelength selectivity and color purity enabled by our q-BIC structure. Second, the grating-based structure highlights the broader extraction bandwidth (~60 nm) and reduced wavelength selectivity when compared with our q-BIC design (bandwidth ~20 nm).

Here, conventional grating-based out-couplers operate based on momentum matching and Bragg diffraction, where the extracted bandwidth tends to be broader due to the resonances involving strong radiative coupling from nanostructures, lack of symmetry-protected confinement, and the relatively low Q-factor nature. As shown in Fig. S36 below, we plotted the planar electric field profiles at the extracted peak wavelength, based on the corresponding grating structure parameters in Fig. S35, revealing stronger

field localization within the nanoblock and across the entire structure.

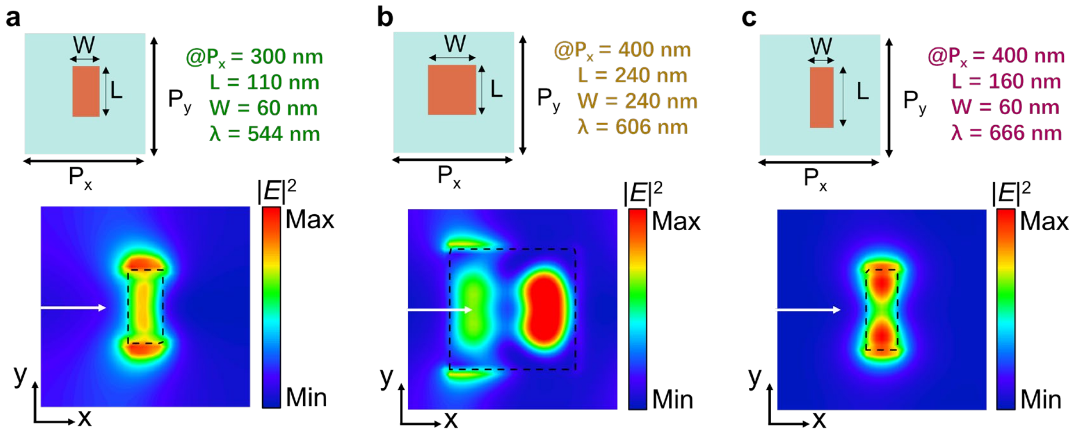

Fig. S36 (a-c) Schematic of three grating-based out-couplers with distinct structure parameters corresponding to Fig. S35, respectively. Bottom: The simulated electric-field intensity ($|E|^2$) distributions in the *xy*-plane for the three cases at the extraction peak wavelengths of (a) 544 nm, (b) 606 nm, and (c) 666 nm.

S19. Comparison of the relative extraction efficiency and the corresponding ratio of Pattern "G" and "R"

Table S2. Comparison of the relative extraction efficiency and the corresponding ratio of Pattern "G" and "R" at 530 nm and 640 nm wavelengths.

	Pattern G	Pattern R
530 nm	0.985	0.025
640 nm	0.051	0.794
E_{530}/E_{640} or E_{640}/E_{530}	19.31	31.76

Table S2 provides the detailed relative extraction efficiency values, along with the corresponding efficiency ratios of Pattern "G" and "R" at 530 nm and 640 nm. Specifically, the relative extraction efficiency ratios of pattern "G" to pattern "R" (or pattern "R" to pattern "G") at 530 nm and 640 nm are 19.31 and 31.76, respectively. Notably, the relative extraction efficiency ratio of pattern "R" at 530 nm and 640 nm surpasses that of pattern "G", indicating a superior color filtering effect for pattern "R", as observed in Figs. 4j and 4m.

S20. Experimental image of "Red Flower & Green Leaf"

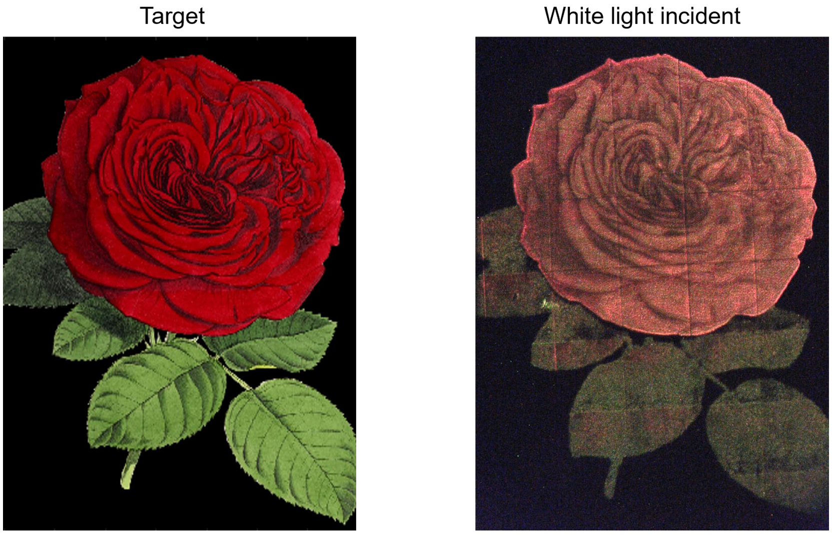

Fig. S37 Experimental image of "Red Flower & Green Leaf" under incidence from the white light source.

S21. The effect of array size on the spatial mapping of color pixels of complex and detailed patterns

In Fig. 3g, we can conclude that more than 20 units along the propagation direction could fully excite the non-local q-BIC modes. Moreover, as the array size decreases, both the q-BIC mode leakage and the peak extraction efficiency deteriorate correspondingly. However, we would like to point out that for complex image display applications, it is not necessarily required to fully excite the q-BIC mode or to perform a parameter-matching design under the condition of maximum peak extraction efficiency. Instead, the detailed image pattern design only requires consideration of the minimum pixel size capable of reliably providing and sustaining the desired color extraction performance.

Based on this, we further performed numerical simulations on on-chip meta-diatomic arrays with finite sizes to systematically evaluate the impact of feasible minimum pixel size on the q-BIC mode and the extraction performance, as shown in Fig. S38. First, we simulated arrays of N × N-unit cells ranging from 2 × 2 to 4 × 4, as shown in Fig. S38a, which illustrates that even when the numbers of unit cells are reduced to 3 × 3, a pronounced extraction peak is still observed, with a peak-to-background contrast exceeding 6. Additionally, we explore possibilities to further reduce the footprint of metasurfaces, while maintaining the desired color extraction performance. As illustrated in Fig. S38b, we investigated the q-BIC mode formation in on-chip meta-

diatomic arrays with N-unit cells in the x-direction (parallel to the guided wave propagation) and only a single row along the y-direction. It can be seen that even a 1 × 7 array exhibits a clear extraction peak with a contrast ratio exceeding 6, although the absolute peak value is relatively low (0.024). Therefore, the array size primarily affects the brightness of colors rather than their saturation. This also means that even with a minimum array size of 1 × 7-unit cells, the pixels retain the ability to extract red color.

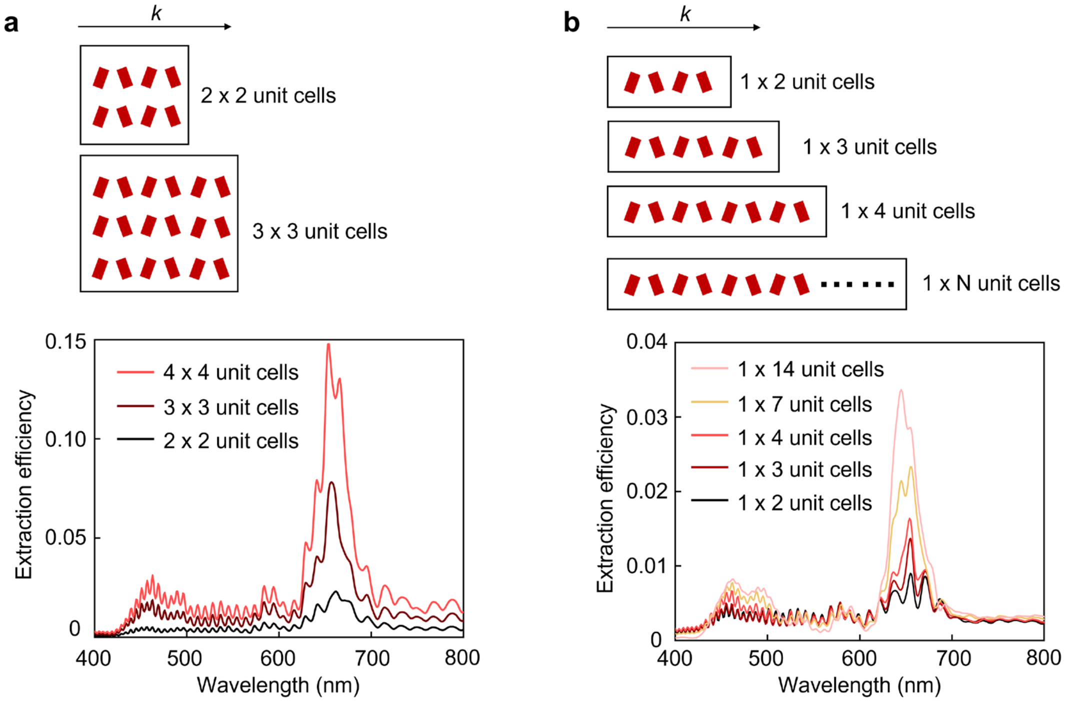

Fig. S38 (a) Extraction spectra of arrays with different sizes, composed of $N \times N$-unit cells. (b) Extraction spectra of arrays composed of $1 \times N$ unit cells with varying N.

Moreover, although the image contains complex details, the design area of the overall image is relatively large (~ 300 × 350 μm^2), which ensures that a sufficient number of pixels are available in detailed regions to maintain the desired color extraction performance. To visualize structure variations at different locations in complex image pattern designs, the mask drawings of the “Red Flower & Green Leaf” pattern in Fig.

S39 illustrate these differences and details.

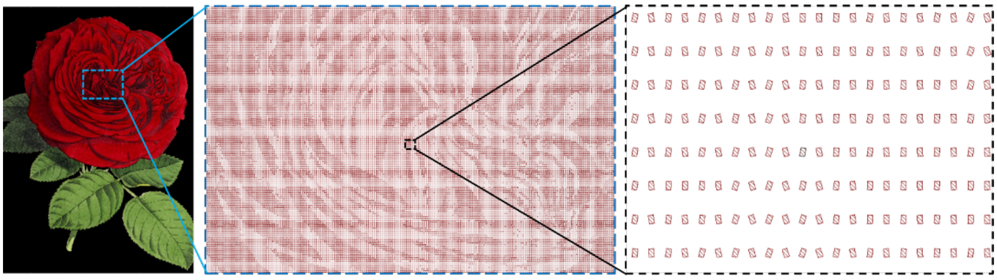

Fig. S39 A partially enlarged schematic diagram of the "Red Flower & Green Leaf" structure array design.

S22. Summary of partial structural parameters, performance, and alternative design solutions

Table S3. Summary of partial structural parameters, performance, and alternative design solutions.

Minimum feasible pixel size	Intensity level	Si meta-atom height	Si_3N_4 waveguide thickness	Other alternative design scheme
(i) 1 x N : 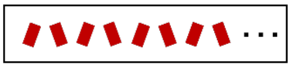 1 x 7 unit cells (ii) N x N : 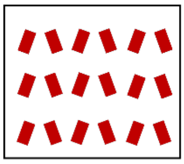 3 x 3 unit cells	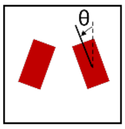 Multiple or continuous steps	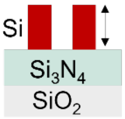 ~ 380 nm	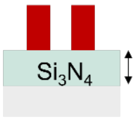 ~ 220 nm ± 30 nm	Etching holes in the waveguide: 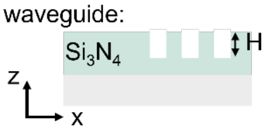 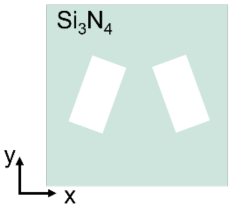

S23. Impact of Q-factor–dependent amplitude modulation on color saturation of extracted light

In our work, amplitude modulation in q-BIC structures is realized by tuning the asymmetry parameter α, defined as $\alpha = \sin\theta$, where θ is the tilting angle of the meta-diatoms. This asymmetry directly governs the radiative Q-factor of the quasi-BIC mode and the bandwidth of the extracted light. As θ increases, the extracted intensity is enhanced, but the Q-factor is reduced, leading to extraction spectral broadening. Both simulated and experimental extraction spectra (Fig. 2h and Fig. 3b in the manuscript) confirm this behavior, with the full-width at half-maximum (FWHM) of the extracted peaks increasing by approximately 5 nm on average as θ varies from 10° to 20°.

Under broadband (white-light) illumination, such Q-factor–dependent amplitude modulation inevitably results in spectral broadening, which influences the color saturation of the extracted light. For example, two structures with identical scaling factors but different tilting angles θ yield the same color, yet differ not only in intensity due to amplitude modulation, but also in color saturation as a result of the different FWHM. This phenomenon reflects an intrinsic characteristic and trade-off of symmetry-protected BIC structures: once perturbed, the bound state gradually radiates into free space, leading to enhanced extraction at the cost of reduced Q-factor and broadened spectra.

While our work demonstrates an important step toward achieving simultaneous amplitude and frequency modulation of extracted guided waves for color routing, we acknowledge that this approach is not yet ideal for applications requiring vivid and finely tunable color display, especially under white-light illumination. On the one hand, the demonstration shown in Fig. 5e ("Demo 3") serves as a proof of concept, showcasing the ability of our on-chip q-BIC-assisted metasurfaces to control both color and intensity simultaneously. On the other hand, our experimental results indicate that the bandwidth variation induced by changes in θ is approximately 5 nm on average. Therefore, although such broadening could, in principle, affect color saturation, we expect the impact on practical display applications to be minor, particularly at relatively high extraction intensities (corresponding to θ values from 10° to 20°), and likely within an acceptable range.

There are several potentially feasible strategies to mitigate the reduction of color saturation caused by amplitude modulation. First, structural optimization, such as introducing higher-order symmetry protection, can maintain a high Q-factor under perturbations while preserving amplitude tunability, thereby suppressing spectral broadening. Second, in imaging and display applications, post-processing methods, e.g., color-compensation algorithms, can enhance visual color saturation. Third, in certain scenarios, illumination with multiple narrowband single-wavelength sources can reduce the influence of spectral broadening on color reproduction. Future work may focus on structural design to realize on-chip narrowband extractors with higher Q-

factors while maintaining amplitude tunability, aiming to further mitigate saturation loss and improve color fidelity in practical applications.
